# EEF1A2 interacts with HSP90AB1 to promote lung adenocarcinoma metastasis via enhancing TGF-β/SMAD signalling

**DOI:** 10.1038/s41416-020-01250-4

**Published:** 2021-01-21

**Authors:** Liqing Jia, Xiaolu Ge, Chao Du, Linna Chen, Yanhong Zhou, Wei Xiong, Juanjuan Xiang, Guiyuan Li, Gaoming Xiao, Li Fang, Zheng Li

**Affiliations:** 1 First Department of thoracic surgery, Hunan Cancer Hospital and the Affiliated Cancer Hospital of Xiangya School of Medicine, Central South University, Changsha, Hunan China; 2 NHC Key Laboratory of Carcinogenesis, Cancer Research Institute, Central South University, Changsha, Hunan China; 3 Shanghai Key Laboratory of Pancreatic Disease, Shanghai General Hospital, Shanghai Jiaotong University, Shanghai, China; 4 The Key Laboratory of Carcinogenesis and Cancer Invasion of the Chinese Ministry of Education, Central South University, Changsha, China

**Keywords:** Cell signalling, Oncogenes

## Abstract

**Background:**

*Eukaryotic protein translation elongation factor 1α2* (*EEF1A2*) is an oncogene that promotes the progression of breast and pancreatic cancer. In this study, we aimed to elucidate the oncogenic function of *EEF1A2* in the metastasis of lung adenocarcinoma (LUAD).

**Methods:**

Immunohistochemistry and western blot were used to study EEF1A2 expression levels in LUAD tissues and cells, respectively. The role of EEF1A2 in LUAD progression were investigated in vitro and in vivo. We identified potential EEF1A2-binding proteins by liquid chromatography-electrospray mass spectrometry (LC-MS)/MS. Protein–protein interactions were determined by immunofluorescence and co-immunoprecipitation (*Co*-IP).

**Results:**

In this study, we report that EEF1A2 mediates the epithelial–mesenchymal transformation (EMT), to promote the metastasis of LUAD cells in vitro and in vivo. Moreover, EEF1A2 interacts with HSP90AB1 to increase TGFβ Receptor (TβR)-I, and TβRII expression, followed by enhanced SMAD3 and pSMAD3 expression and nuclear localisation, which promotes the EMT of LUAD cells. Overexpression of EEF1A2 in cancer tissues is associated with poor prognosis and short survival of patients with LUAD.

**Conclusions:**

These findings underscore the molecular functions of EEF1A2 in LUAD metastasis and indicate that EEF1A2 represents a promising target in the treatment of aggressive LUAD.

## Background

Lung cancer is the most prevalent malignancy, causing 1.77 million deaths worldwide annually.^1^ Lung cancer is divided into two major categories, the non-small cell lung cancer (NSCLC), and small cell lung cancer (SCLC). NSCLC includes lung adenocarcinoma (LUAD), and squamous cell carcinoma (SCC), which accounts for 80–85% of all lung cancer cases.^2^ Although earlier reports have confirmed the expression characteristics and corresponding therapeutic strategies targeting *KRAS*, and *EGFR* mutations, and the *EML4-ALK* fusion gene in LUAD, the 5-year overall survival rate of lung adenocarcinoma patients remains low at 18%.^3–7^ In particular, only 2–8% of patients with late stage lung adenocarcinoma can live up to 5 years.^8^ Therefore, an improved understanding of the key underlying metastatic mechanisms of LUAD progression is required.

Recent studies have shown that several housekeeping proteins contribute to enhance epithelial–mesenchymal transformation (EMT) and oncogenic transformation.^9–11^ β2-microglobulin (β2-M), a well-known heterodimeric cell surface and housekeeping protein presents antigenic peptides to cytotoxic T cells.^12,13^ Additionally, β2-M interacts with receptor protein hemochromatosis to modulate EMT and metastasis of cancer cells.^14^ Recently, an increasing number of studies have confirmed that translation factors had a positive role in regulating cytoskeleton altering signalling pathways.^15–17^ *Eukaryotic protein translation elongation factor 1α2* (*EEF1A2*) belongs to the eEF1A family and localises to chromosome 20q13.3. It consists of three domains, which predominantly deliver the aminoacylated-tRNA to the ribosome A site for decoding of mRNA by codon–anticodon interactions during protein synthesis.^18,19^ Additionally, *EEF1A2* regulates myotube survival by protecting them from undergoing apoptosis.^20^ Moreover, *EEFlA2* was reported as a putative oncogene due to its high expression in breast, ovarian, lung and liver cancers.^21–24^ A previous report showed that EEF1A2-induced Akt-dependent actin remodelling, and enhanced the invasion of breast cancer cells.^25^ Additionally, EEF1A2 promotes pancreatic cancer metastasis through Akt-dependent upregulation of MMP-9 expression.^26,27^

In lung cancer cells, a comparative genomic hybridisation (CGH) array identified DNA gain at 20q13 region, while fluorescence in situ hybridisation (FISH) analysis confirmed DNA copy number increase of EEF1A2.^28^ EEF1A2 enhanced Gefitinib resistance by preventing AKT phosphorylation for KRAS-mutated NSCLC.^29^ Li et al. showed that the DNA copy number, mRNA, and protein expression of EEF1A2 were increased in lung cancer cells using integrated omics analysis. Additionally, EEF1A2 overexpression has been correlated with tumour stage in NSCLC patients.^30^ However, the function and molecular mechanism of EEF1A2 in promoting development and metastasis of LUAD has not been identified. In this study, we aimed to elucidate the oncogenic function and mechanisms of *EEF1A2* in the proliferation and metastasis of LUAD.

## Methods

### Clinical specimens and bioinformatics analysis

To elucidate the effect of upregulated EEF1A2 on promotion of metastasis in LUAD patients, a tissue array consisting of 78 para-carcinoma, 78 primary LUAD and four metastatic LUAD tissues were subjected to immunohistochemistry (IHC) analysis (Supplementary Table 1). All specimens in the tissue array were verified by a clinical pathologist. Datasets GSE37745 (consisting of 106 LUAD tissue samples) and GSE19188 (including 65 normal lung tissue samples, and 45 LUAD tissue samples) were obtained from the public database Gene Expression Omnibus (GEO) (http://www.ncbi.nlm.nih.gov/geo/) and reanalysed to elucidate the correlation between EEF1A2, HSP90AB1 expression level and survival time of LUAD patients.^31,32^ The GSE19188 dataset provided expression data for EEF1A2 and HSP90AB1 for comparison between LUAD and normal lung tissue samples. The GSE37745 dataset was analysed using the Kaplan–Meier method to create a survival curve of EEF1A2 and HSP90AB1 in LUAD tissue samples.

### Cell lines

To investigate the function and molecular mechanism of EEF1A2 in LUAD, we used A549 and PC9 cells. Immortalised normal bronchial epithelial cells, HBE and LUAD cell lines, A549 and PC9 were cultured in RPMI-1640 medium supplemented with 10% FBS. The cells were grown at 37 °C in a humidified atmosphere with 5% CO_2_. Growing cells were tested for mycoplasma contamination using polymerase chain reaction (PCR).

### Immunohistochemistry and antibodies

Following deparaffinisation and rehydration using regular graded alcohol and quenching with hydrogen peroxide, tissue sections on glass slides were incubated with 0.01 M citrate buffer for antigen retrieval. The slides were incubated with primary antibody at 4 °C overnight. The sections were washed with phosphate buffer solution (PBS) three times, then stained with polymerised horseradish peroxidase and anti-rabbit IgG (Cell Signaling Technology, USA) for 1 h. Finally, the slides were washed, stained using 3’-diaminobenzidine hydrochloride as the chromogen, and counterstained with haematoxylin. Positive labelling in the cell nucleus was visualised and images were captured using a stereomicroscope (OLYMPUS BX-51, Tokyo, Japan). EEF1A2 expression was evaluated based on the staining intensity and area, where H score = 1 × (% weak) + 2 × (% moderate) + 3 × (% intense); range = 0–300.^33^ EEF1A2 and HSP90AB1 antibodies were purchased from ABclonal (Wuhan, China) and Affinity (Cincinnati, OH, USA), respectively. Antibody information is detailed in Supplementary Table 2.

### SiRNAs, plasmids and transduction

EEF1A2 and HSP90AB1 siRNAs were obtained from Ribobio Biological Technology (Guangzhou, China). pEnter-EEF1A2 plasmid with c-terminal Flag and His tag was purchased from ViGene Biosciences (Jinan, China). EEF1A2-Flag and HSP90AB1-Myc truncation mutants were generated using the pcDNA3.1(+) vector backbone. These plasmids were co-transfected into HEK293 cells to investigate the binding domains of EEF1A2 and HSP90AB1. To establish stable EEF1A2 overexpression and knockdown cells, the EEF1A2 open reading frame sequence was cloned into the lentiviral plasmid pCDH-green fluorescent protein (GFP), and shRNAs targeting EEF1A2 were constructed using the pLVTH-GFP vector. The vectors were co-transfected with psPAX2 and pMD2.G and the cells were enriched based on their GFP expression using flow cytometry. To perform live monitoring of tumour cells in mice, luciferase was stably expressed in sh-EEF1A2 cells using lentiviral plasmids, and enriched by blasticidin screening (YESEN, Shanghai, China). The sequence of EEF1A2 siRNAs, shRNAs and HSP90AB1 siRNA are shown in Supplementary Table 3.

### In vivo experiments

To investigate the function of EEF1A2 in vivo, a total of 26 4-week-old mice (18–25 g, Balb/c nude) were purchased from Hunan SJA Laboratory Animal Company and fed a rodent diet at the Experimental Animal Center of Central South University (Changsha, China). Mice were subjected to 12-h dark, and 12-h light conditions and a standard temperature of 23 °C and humidity of 48%. A subcutaneous tumour mouse model was used to confirm the role of EEF1A2 in tumorigenesis of LUAD. Sixteen female Balb/c nude mice were randomly and equally divided into two groups: EEF1A2 knockdown, and control groups. The sh-EEF1A2_001 and negative control cells in the logarithmic growth phase were collected and washed once with PBS. The cells were resuspended at 1 × 10^7^ cells/mL, and subcutaneously injected (200 μL) into the right anterior axillary of the mice. The tumours were measured daily after injection, and growth curves were plotted for each group. The maximum (length) and minimum diameter (width) of the tumour in each mouse were measured using a Vernier calliper to calculate the tumour volume using the formula: Volume = length × width^2^ × 0.52. All the mice were sacrificed using excess carbon dioxide (25% chamber volume per minute) 28 days after-inoculation. Tumours were isolated, photographed and weighed.

A metastatic tumour mouse model was used to confirm the prometastatic potential of EEF1A2. Ten female Balb/c nude mice were randomly and equally divided into the EEF1A2 knockdown, and control groups. Sh-EEF1A2_001 or the negative control A549 cells were collected and resuspended at 2 × 10^7^ cells/mL. The cell suspension (200 μL) was injected into the tail veins of Balb/c nude mice using a 1 mL syringe (4 × 10^6^ cells per mouse). The metastatic stage was monitored 7–8 weeks post-injection when all mice were under inhalation anaesthesia with 2% isoflurane, and luciferin substrate (150 mg/kg) was injected and visualised using an in vivo luminescence imaging system (IVIS, PerkinElmer, MA, USA). The mice were sacrificed using excess carbon dioxide (25% chamber volume per minute) 8 weeks post-injection, and the lungs and lymph nodes were removed for further analysis. Haematoxylin and Eosin staining was used to observe the metastasis of LUAD cells, to examine the cellular morphology, and record the number of metastatic tumour foci in the lung and lymph nodes using a stereomicroscope.

### Immunoprecipitation and liquid chromatography mass spectrometry (LC-MS)/MS analysis

HEK293 cells (5 × 10^6^) were lysed using cell lysis buffer for immunoblotting and immunoprecipitation (P0013, Beyotime, Shanghai, China) according to the manufacturer’s protocol. Following centrifugation at 12,000 × *g* for 20 min at 4 °C, the supernatant was incubated with primary antibodies overnight at 4 °C, followed by incubation with protein A + G Sepharose (30 μL) (Millipore, USA) for an additional 4 h. The beads were washed five times with ice-cold lysis buffer. The bound proteins were eluted from the beads with SDS-loading buffer, and separated on SDS-PAGE, followed by LC-MS/MS analysis, and immunoblotting. Briefly, the gel pieces were subjected to in-gel tryptic digestion after removing the SDS. Then, the peptide was desalted using trifluoroacetic acid (Sigma, USA) on a C18 elution plate (Sigma, USA). Peptide analysis was carried out using an Ultimate 3000 nano HPLC system, and electrospray ionisation-tandem mass spectrometer (Thermo Fisher Scientific, USA). Data were processed, searched and quantified using Proteome Discoverer 1.4 software (Thermo Scientific, Waltham, MA, USA), using the default settings and employing the Human Uniprot database (Proteome ID: UP000005640). To find proteins that interact with EEF1A2, further bioinformatics analysis of all the protein data was performed using the Functional Enrichment analysis tool (FunRich Version 3.0). All proteins were listed in Supplemental Tables 4 and 5.

### Immunofluorescence microscopy

A549 cells were transfected with si-EEF1A2 or the negative control, or Flag-EEF1A2, and Myc-HSP90AB1 for 48 h. The fluorescence was directly visualised and recorded. The cells were cultured on glass coverslips in a six-well plate. Thereafter, the cells were fixed with 4% paraformaldehyde in PBS, permeabilised by 0.1% Triton X-100, followed by washing in PBS. Phalloidin (P2141, Sigma, USA) in TBST was added onto the coverslips and incubated for 1 h. Thereafter, anti-Flag (1:500) or anti-Myc antibodies (1:100) was added and incubated overnight at 4 °C. Subsequently, the coverslips were incubated with the corresponding Alexa Fluor 488 (SA00006-1, Proteintech, China) or Alexa Fluor 594 (SA00006-4, Proteintech, China) secondary antibodies. Cellular nuclei were stained with DAPI (Beyotime Biotechnology, Shanghai, China).

### Statistical analysis

Statistical analysis of cell proliferation, colon formation, cell invasion and metastasis and animal tumour weight were performed using the SPSS software (version 20.0, IBM, Chicago, USA). All statistical calculations and plotting of graphs were performed using the GraphPad Prism5 software (GraphPad Software LLC, San Diego, USA). Significant differences between two groups of data in the invasion, wound healing, and colony formation assays were evaluated by using the Student’s *t*-test. One-way analysis of variance (ANOVA) was used for more than two groups. Overall survival (OS) or relapse-free survival (RFS) for different levels of EEF1A2 or HSP90AB1 signature was calculated using the Kaplan–Meier method. Statistical significance was determined using the Student’s *t*-test, Spearman correlation coefficients test, Kaplan–Meier method, or Fisher’s exact test. For all statistical tests, *p* < 0.05 was considered statistically significant.

## Results

### EEF1A2 overexpression in LUAD tissues and cells is strongly correlated with poor prognosis

**Fig. 1:**
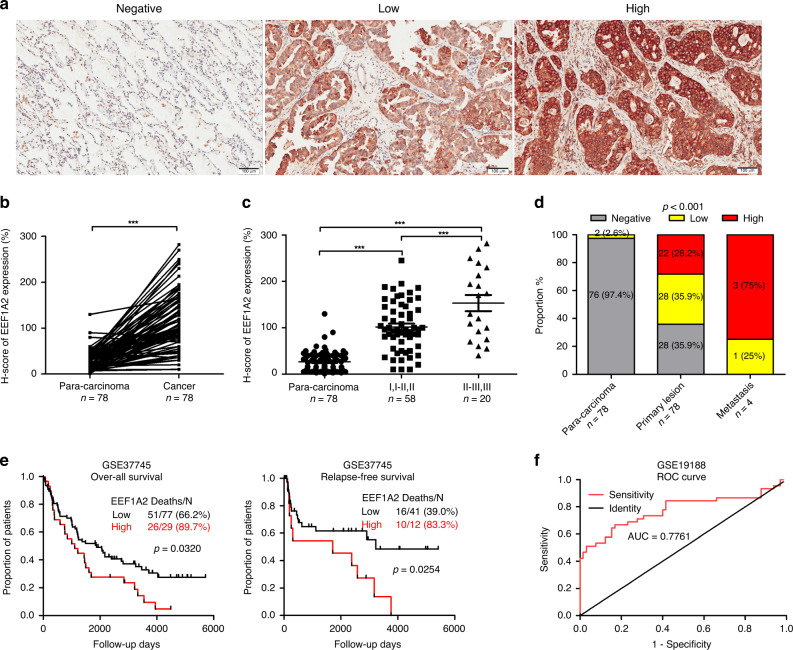
Expression of EEF1A2 and clinical significance in lung adenocarcinoma (LUAD). EEF1A2 expression was detected by immunohistochemistry (IHC) of paraffin-embedded tissues of LUAD patients. **a** Representative images were shown for negative, low or high expression of EEF1A2 staining. **b** Quantitative analysis of EEF1A2 expression in 78 paired LUAD tissues (T) and adjacent tissues (N). **c** EEF1A2 expression was positively related to the clinical pathologic stage (Para-carcinoma, *n* = 78; I, I–II, II *n* = 58; II–III, III *n* = 20). **d** The distribution of EEF1A2 expression levels in different tissues. (para-carcinoma *n* = 78, primary lesion *n* = 78 and metastasis *n* = 4). **e** Kaplan–Meier curve analysis of overall survival (OS) and relapse-free survival (RFS) of LUAD patients according to different EEF1A2 expression levels. **f** Receiver operating characteristic (ROC) curves of EEF1A2 for the diagnosis of LUAD. (Healthy, *n* = 65; LUAD patients, *n* = 45). (AUC = 0.7761, 95% CI: 0.6778 to 0.8743) *p < 0.05, **p < 0.01, and ***p < 0.001, NS no statistical significance.

To determine the clinical significance of the EEF1A2 in patients with LUAD, immunohistochemical staining was used to detect EEF1A2 in tissue microarrays (TMAs) consisting of 78 pairs of cancer samples compared with their corresponding adjacent tissues. Compared to the para-carcinoma tissues, tumour tissues exhibited strong expression of EEF1A2 (Fig. 1a, b). Notably, EEF1A2 expression was further enhanced in advanced-stage and distant-metastatic tumour tissues (Fig. 1c, d). Additionally, a higher EEF1A2 expression level was associated with poorer OS and RFS (Fig. 1e). As shown in Fig. 1f, the area under the receiver operating characteristic (ROC) curve (AUC value) of EEF1A2 for the diagnosis of LUAD was 0.7761. These results suggested that EEF1A2 might be an oncoprotein involved in the progression of LUAD, and an early diagnosis and prognostic factor thereof.

### EEF1A2 promotes the development of malignant phenotypes of LUAD cells

**Fig. 2:**
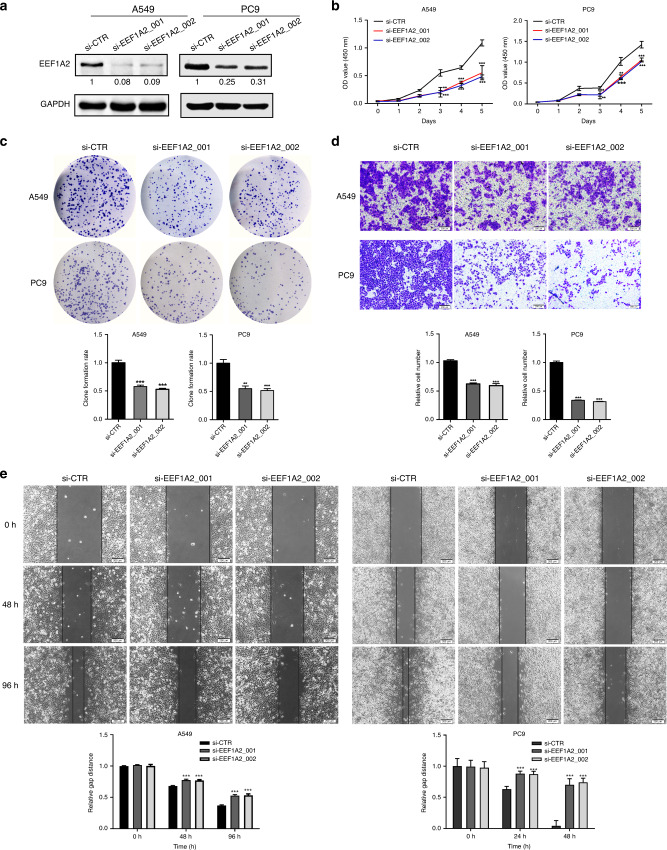
Silencing of EEF1A2 decreases the proliferation, invasion, and migration abilities of A549 cells. **a** The expression of EEF1A2 after si-EEF1A2s transfected into A549 and PC9 cells. **b** Cell count kit 8 (CCK8) assay and **c** colony formation after transfected with si-EEF1A2s in A549 cells and PC9 cells (*n* = 3). **d** Trans-well assay was performed as silenced EEF1A2 in A549 and PC9 (*n* = 3). Scale bar, 200 μm. Number of invasive cells was shown as mean ± SEM. **e** The migration ability was analysed using a wound-healing assay in EEF1A2-knockdown A549 and PC9 cells (*n* = 3). Scale bar, 200 μm. The distance of the gap was shown as mean ± SEM. *p < 0.05, ** *p* < 0.01, *** *p* < 0.001, NS no statistical significance.

The expression of EEF1A2 was higher in A549 and PC9 cells than in HBE cells. EEF1A1 protein shares 96% homology in amino acid sequence with EEF1A2, although no expression level changes were noted in these cells (Fig. S1A). To examine the function of EEF1A2 in LUAD tumorigenesis, siRNAs targeting EEF1A2 were transfected into A549 and PC9 cells to reduce the expression of EEF1A2 (Fig. 2a). The cell counting kit 8 (CCK8) assay demonstrated that reduction of EEF1A2 decreased the proliferation of A549 and PC9 cells (Fig. 2b). The colony formation assay confirmed that the downregulation of EEF1A2 led to a considerable reduction in the number of colonies compared to the control (Fig. 2c). As clinical data showed high expression levels of EEF1A2 in metastatic LUAD tumour tissues, we investigated the effect of EEF1A2 on metastatic ability of LUAD cells. Cell invasion and wound-healing assays showed that the invasive and migration ability was repressed in A549 and PC9 EEF1A2-knockdown cells (Fig. 2d, e). In contrast, EEF1A2 overexpression in A549 and PC9 cells promoted cellular proliferation, invasiveness, and migration (Fig. S1B–F).

### EEF1A2 promotes LUAD cell proliferation and metastases in vivo

**Fig. 3:**
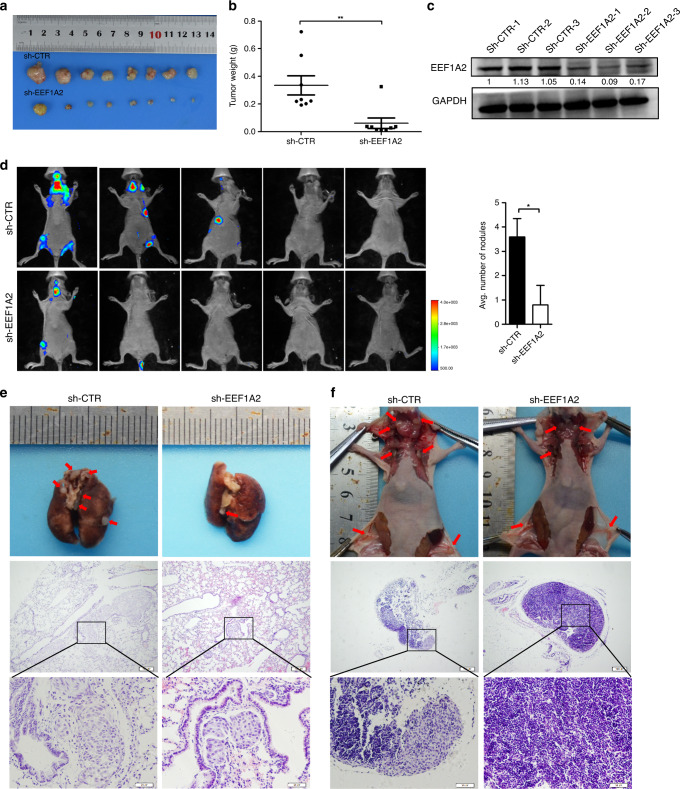
EEF1A2 silencing inhibits tumour growth and metastasis in vivo. **a** Balb/c nude mice were subcutaneously injected with cells stably-silenced EEF1A2 (sh-EEF1A2_001) or control A549 cells (sh-CTR). Gross pathology of the subcutaneously xenograft immediately after resection. *n* = 8. **b** Tumour formation was monitored at the indicated intervals, and tumour weights were recorded. Data are shown as mean ± SEM; *n* = 8, two-tailed Student’s *t*-test. **c** The EEF1A2 expression level of the control and sh-EEF1A2_001 group. **d** Luciferase-labelled sh-CTR or sh-EEF1A2_001 A549 cells were intravenously injected into nude mice. Bioluminescence was examined after 8 weeks. The number of metastatic nodules in the lungs was shown as mean ± SEM (*n* = 5), using a two-tailed Student’s *t*-test. **e**, **f** Metastatic lung (**e**), and lymph nodes (**f**) in mice were presented and confirmed by Haematoxylin and Eosin staining (scale bar, 200 and 50 μm, respectively). *p < 0.05, **p < 0.01, and ***p < 0.001, NS no statistical significance.

To elucidate the function of EEF1A2 in vivo, we designed lentiviral vectors expressing EEF1A2 shRNAs or control shRNA with luciferase expression. In sh-EEF1A2_001 A549 cells, the expression level of EEF1A2 decreased by ~90% (Fig. S2A). The cell invasion assay showed that EEF1A2 knockdown by shRNAs effectively reduced the invasive ability of A549 cells (Fig. S2B). Hence, Sh-EEF1A2_001 A549 cell line was selected for the animal experiments. As expected, EEF1A2 knockdown delayed tumour growth in mice xenografts (Figs. 3a, b and S2C). Additionally, western blot results verified that EEF1A2 protein expression decreased in tumour tissues of the sh-EEF1A2 group (Fig. 2c). Thereafter, we tested the effect of EEF1A2 on metastasis in vivo using a tail vein injection model. As shown in Fig. 3d, the level of tumour burden was decreased in sh-EEF1A2_001-A549 cells group. Fewer lung and lymph metastatic nodules were detected compared to the control group (Fig. 3e, f). Collectively, these data demonstrated that EEF1A2 knockdown inhibited lung tumorigenesis and metastasis in vivo.

### EEF1A2 promotes EMT by enhancing TGF-β/SMAD signalling

**Fig. 4:**
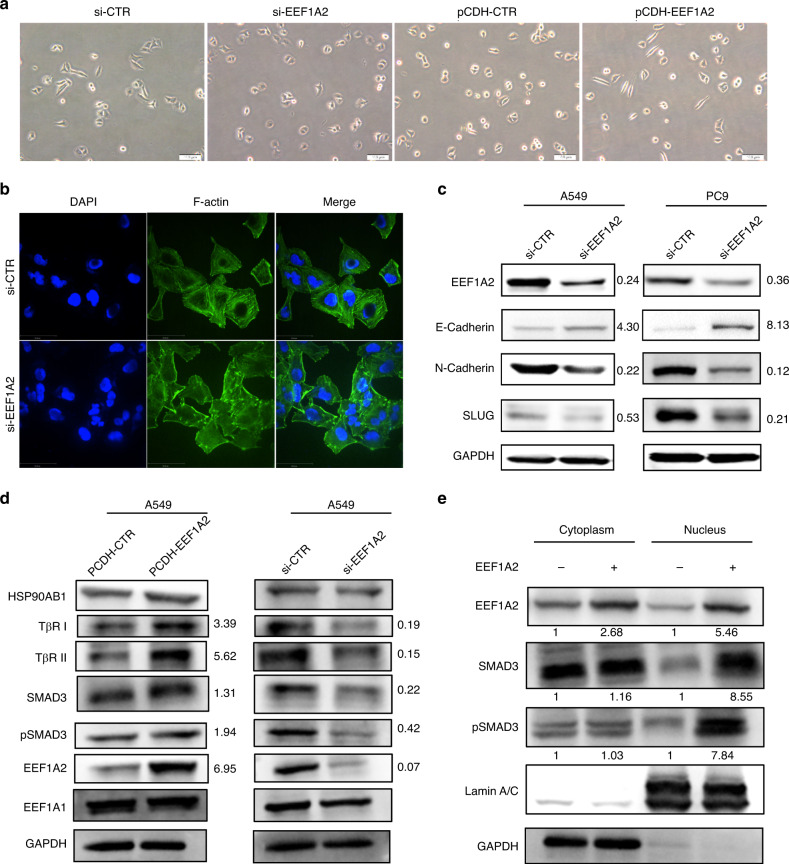
EEF1A2 induces EMT processes via upregulation of TβRI, TβRII and pSMAD3 nuclear expression. **a** Influence of EEF1A2 expression on cell morphology. Images of A549 cells after EEF1A2 silenced or overexpressed. Scale bar = 100 μm. **b** Immunofluorescence staining of F-actin with phalloidin in A549 cells after EEF1A2 knockdown. Scale bar = 50 µm. **c** Western blot analysis of EMT marker (E-Cadherin, N-Cadherin and SLUG) protein expression levels in A549 and PC9 cells after EEF1A2 silencing. **d** Western blot analysis of the TβRI, TβRII, HSP90AB1, EEF1A1, phosphorylation and total SMAD3 protein expression levels in A549 cells with EEF1A2 overexpression or knockdown. **e** Cytoplasmic and nuclear fractions of A549 cells with or without EEF1A2 overexpression were isolated, and western blot analysis was performed with anti-pSMAD3, total SMAD3, GAPDH and Lamin A/C antibodies.

As shown in Fig. 4a, EEF1A2 knockdown changed the morphology of A549 cells from a mesenchymal-like shape to a cobblestone appearance. Furthermore, cytoskeletal change detected by FITC-phalloidin (green), showed that EEF1A2 knockdown induced disorderly microfilaments and disruption of stress fibres (Fig. 4b). We hypothesised that EMT may be involved in the EEF1A2-induced changes in cell morphology. Compared to the control cells, the expression of epithelial-like marker (E-Cadherin) was upregulated, and expression of mesothelial-related proteins (N-Cadherin and SLUG) was decreased in the A549 and PC9 cells treated with si-EEF1A2 (Fig. 4c). These results suggested that EMT activity may be involved in the function of EEF1A2 to promote invasion and metastasis of LUAD cells.

TGF-β/SMAD signalling is an important inducer of EMT in various cancers.^34–36^ We found that EEF1A2 overexpression or silencing caused a change in the expression of two key receptors in the TGF-β signalling pathway, TGF-β receptor type I (TβR I) and type II (TβR II). This was accompanied by the change in the SMAD3 and pSMAD3 expression levels (Fig. 4d). Furthermore, we detected changes in SMAD3 and pSMAD3 expression in the cytoplasm and nucleus, following EEF1A2 overexpression. Additionally, there was no significant change in the expression of SMAD3 and pSMAD3 in the cytoplasm of A549 cells, whereas that in the nucleus increased (Fig. 4e). These results suggested that EEF1A2 could promote EMT through modulation of the TGF-β receptor and nuclear expression of SMAD3 proteins.

### EEF1A2 regulates TGF-β/SMAD signalling and EMT by interacting with HSP90AB1

**Fig. 5:**
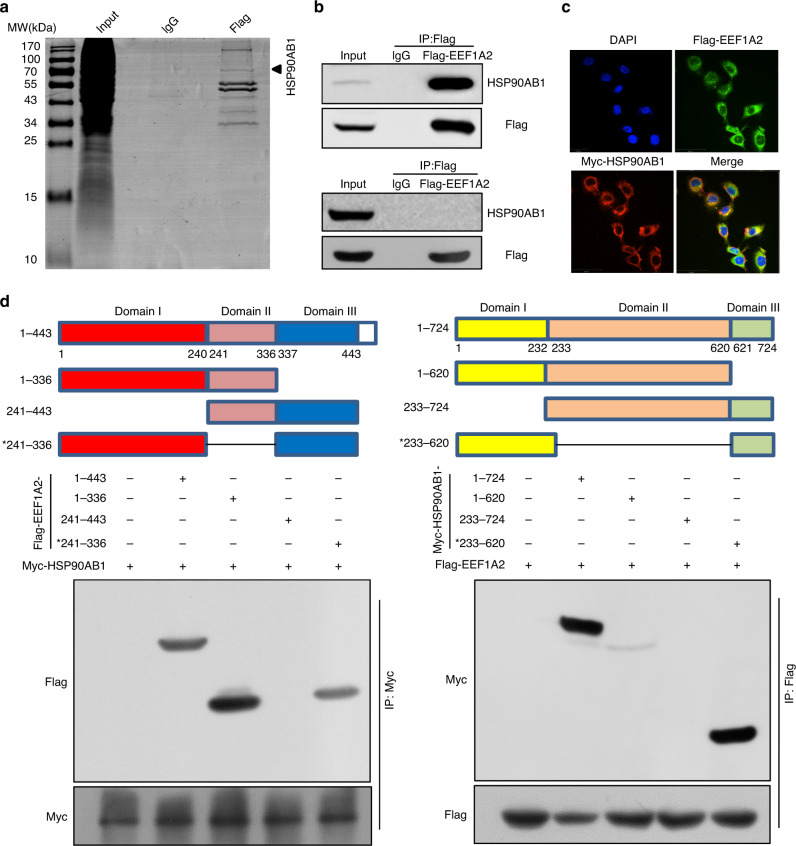
EEF1A2 interacts with the ATP-binding domain of HSP90AB1. **a** Cell lysates from 293 T cells transfected with Flag-EEF1A2 were immunoprecipitated using anti-Flag agarose and visualised by Coomassie blue staining. Interacting proteins were identified by liquid chromatography mass spectrometry (LC-MS)/MS. **b** A549 cells were transfected with Flag-EEF1A2, and the proteins were immunoprecipitated with Flag monoclonal antibody. The HSP90AB1 and HSP90AA1 were detected by immunoblotting. **c** Confocal images showing expression of Flag-tagged EEF1A2 (green) and Myc-tagged HSP90AB1 (red) in A549 cells. Red and green images were acquired sequentially. Scale bar = 50 µm. **d** Domain organisation and co-immunoprecipitation analysis of three Flag-EEF1A2 truncation fragments (left), and three Myc-HSP90AB1 truncation fragments (right).

To gain further insights into the molecular mechanism of EEF1A2, we used Flag IP-pull-down followed by LC-MS/MS to identify potential EEF1A2-binding proteins (Fig. 5a). A total of 127 proteins shared by A549 and PC9 cells that interacted with EEF1A2 were identified. However, we did not identify the SMADs amongst these proteins (Supplementary Tables 4 and 5). Additionally, we verified that EEF1G could interact with EEF1A2 as previously reported,^37^ indicating the reliability of the LC-MS/MS analysis (Fig. S3A). Notably, the immunoprecipitation assay showed that EEF1A2 could be precipitated with HSP90AB1 and XRCC6, although not with HSP90AA1, which was found in LC-MS/MS analysis (Fig. 5b and Fig. S3A & B). Previous studies have shown that inhibition of heat shock protein 90 (HSP90) function leads to the ubiquitination and degradation of TβR.^38^ Lee et al. reported that inhibition of HSP90AB1 decreased nuclear localisation and phosphorylation of SMAD3 in Mv1Lu cells.^39^ Therefore, we suggested that EEF1A2 may regulate TGF-β/SMAD3 signalling through HSP90AB1 in LUAD cells.

Immunofluorescence staining of EEF1A2 and HSP90AB1, followed by confocal microscopy revealed strong staining in the cytoplasm and scattered staining in the nuclei. Marked co-staining of EEF1A2 and HSP90AB1 was visualised in the cytoplasm (Fig. 5c). Neither si-EEF1A2 nor si-HSP90AB1 did alter the position of their co-location in the cytoplasm (Fig. S3D). The EEF1A2 protein consists of three domains, namely domain I, domain II, domain III. Domain I consists of 1–240 amino acids that structured in a Rossmann-fold topology. Domain II (241–336 aa) and domain III (337–443 aa) span over beta strands and each domain contains two beta sheets that form the beta barrel.^18^ HSP90AB1 consists of three major structural domains: the nucleotide binding domain (domain I, 1–232 aa) located in the N-terminal portion, which contains the ATP-binding site; the client binding domain (domain II, 233–620 aa) in the centre; and a C-terminal domain (domain III, 621–724 aa) responsible for HSP90AB1 dimerisation.^40^ We generated various EEF1A2-Flag and HSP90AB1-Myc deletion constructs to investigate the domain responsible for binding. The pull-down assay showed that Flag-tagged EEF1A2 domain I (1–240 aa) proteins could bind to HSP90AB1. Additionally, the HSP90AB1 domain I (1–232 aa) is predominantly responsible for interaction with EEF1A2 (Figs. 5d and S3C).

**Fig. 6:**
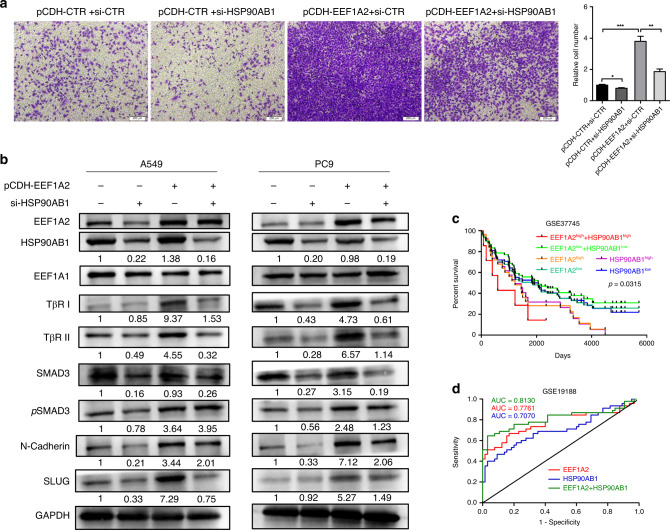
EEF1A2 regulates TGF-β/SMAD signalling based on HSP90AB1. **a** HSP90AB1 silencing inhibited the invasion potential of A549 cells induced by EEF1A2 overexpression. Data are shown as mean ± SEM; *n* = 3 independent experiments, two-tailed Student’s *t*-test. **b** A549 and PC9 cells were co-transfected with PCDH-EEF1A2, and si-HSP90AB1. The protein expression levels of HSP90AB1, EEF1A1, TβRI, TβRII, SMAD3, pSMAD3, N-cadherin and SLUG were detected using western blot analysis. **c** Overall survival according to Kaplan–Meier analysis showed the survival rate of LUAD patients with EEF1A2 overexpression, and HSP90AB1 overexpression separately or combined. **d** The ROC curves of EEF1A2 was combined with HSP90AB1 for the diagnosis of LUAD. (AUC = 0.8130, 95% CI: 0.7188–0.9072). *p < 0.05, **p < 0.01, and ***p < 0.001, NS no statistical significance.

The expression of HSP90AB1 was higher in A549 and PC9 cells than in HBE cells (Fig. S4A). HSP90AB1 knockdown attenuated the invasive ability of LUAD in pCDH-EEF1A2 A549 cells (Fig. 6a). In A549 and PC9, HSP90AB1 knockdown could partially reverse the increase of TβRI, TβRII, SMAD3, pSMAD3, N-cadherin and SLUG expression levels in LUAD cells, induced by EEF1A2 overexpression (Figs. 6b and S4B). Overall, these data suggest that EEF1A2 interacts with HSP90AB1, and regulates TGF-β signal transduction, thus affecting the migration of LUAD cells.

Overexpression of HSP90AB1 also correlated with poor prognosis in LUAD (Fig. S4C, D). We calculated the OS for LUAD patients using the combined index of EEF1A2 and HSP90AB1 expression. The results showed that a combination of high EEF1A2 and HSP90AB1 were significantly associated with the worst prognosis in LUAD patients (Fig. 6c). As depicted in Fig. 6d, the AUC of EEF1A2 and HSP90AB1 for diagnosis was 0.7761 and 0.7070, respectively. The combination of EEF1A2 and HSP90AB1 analysed by logistic regression showed better discrimination as the AUC was 0.8130. Hence, the combination of EEF1A2 and HSP90AB1 was a useful parameter for the diagnosis of LUAD patients.

## Discussion

The present study identified that the expression of EEF1A2 increased in LUAD compared to para-carcinoma tissues and was similarly higher in metastatic tissues compared to primary tumour tissues. These results support that of previous studies, which showed that EEF1A2 was strongly expressed in LUAD cells and tumour tissues.^28,30^ Moreover, we discovered that upregulated expression of EEF1A2 positively correlated with the tumour stage and OS in LUAD patients. Furthermore, we demonstrated that EEF1A2 overexpression promoted the proliferation, migration and invasive abilities of LUAD cells in vitro. Subcutaneous and metastatic tumour mouse models revealed that *EEF1A2* was a putative oncogene that promoted progression of LUAD in vivo.

Epithelial–mesenchymal transformation is one of the fundamental processes that contribute to tumour progression, particularly metastatic spread and colonisation of cancer.^41,42^ During EMT, nonmotile polarised epithelial cells are converted into motile nonpolarised invasive mesenchymal cells as a result of a series of EMT specific transcription factor (ZEB1/2, SNAIL, SLUG and others) changes.^43–45^ Our results showed that EEF1A2 silencing induced epithelial-like morphological features in LUAD cells, decreased expression of mesenchymal marker N-Cadherin and SLUG and upregulated epithelial marker E-Cadherin. TGF-β signalling is a potent initiator of EMT through activating and transforming phosphorylated SMAD proteins in the nucleus, and inducing cell motility processes.^38,46,47^ We observed that EEF1A2 enhanced TβRI and TβRII expression and pSMAD3 nuclear expression, which induced expression of downstream EMT-related transcription factors, and promoted metastasis of LUAD cells.

To clarify the molecular mechanism of EEF1A2 functionality, a pull-down experiment was conducted in A549 and PC9 cells, and potential interaction protein partners of EEF1A2 were identified by LC-MS. EEF1A2 interaction proteins included EEF1B2 and EEF1G, and new potential partners, such as HSP90AA1, HSP90AB1 and XRCC6, among them. Notably, EEF1A2 selectively combined with XRCC6 and HSP90AB1, although not with HSP90AA1. XRCC6 interacts with BAX to prevent BAX-induced apoptosis in LUAD.^48^ Inhibition of HSP90 functionality using small molecule inhibitor 17-allylamino-17-demethoxy-geldanamycin (17AAG) increased TβRI/II ubiquitylation and degradation, and inhibited TGFβ-induced phosphorylation and nuclear transport of endogenous SMAD2/3 in melanoma cells.^38^ The present study filled in the gaps of these previous reports as we elucidated that EEF1A2 regulated TGF-β/SMAD signalling and EMT through HSP90AB1. Furthermore, we demonstrated binding of the EEF1A2 Rossmann-fold topology domain to the ATP-binding domain of HSP90AB1. HSP90AB1 belongs to the classical molecular chaperone family of HSP90 that is associated with the prognosis and evolution of neoplasia, similar to other HSPs proteins in cancer.^49^ Upregulation of HSP90AB1 has been reported in liver and gastric cancer, which activates VEGFR transcription and stabilises LRP5 through activation of the AKT and Wnt/β-catenin signalling pathway, leading to carcinogenesis and invasion.^50,51^ The present study showed that downregulated expression of HSP90AB1 decreased TβRI, TβRII, SMAD3, pSMAD3, N-cadherin and SLUG expression levels, which was enhanced by the overexpression of EEF1A2. Similarly, downregulation of HSP90AB1 inhibited cell invasion ability, which was elevated by EEF1A2. Thus, EEF1A2 interacted with HSP90AB1 to enhance TGF-β/SMAD signalling activity to promote EMT of LUAD cells.

EEF1A2 is a potential target for cancer treatment. Plitidepsin is an antitumour agent of marine origin targeting *EEF1A2* that has completed Phase 3 clinical trials for multiple myelomas.^52,53^ Metarrestin interacts with EEF1A2 to disrupt the perinucleolar compartment (PNC), and inhibits invasive ability of pancreatic cancer cells.^54^ Overexpression of eEF1A2 was associated with metastasis and poor prognosis of LUAD patients; hence, EEF1A2 is a potential target for the treatment of aggressive LUAD.

## Conclusions

In conclusion, the present study identified a novel functional role for EEF1A2 in LUAD metastasis in vitro and in vivo. EEF1A2 is a new oncogenic partner of HSP90AB1 due to its binding with the nucleotide binding domain of HSP90AB1. Furthermore, EEF1A2 regulated TβRI and TβRII expression and SMAD3 nuclear expression based on HSP90AB1 in LUAD cells. Overall, our results revealed that EEF1A2 interacted with HSP90AB1 to activate SMAD3 signalling and promote tumour metastasis. Therefore, EEF1A2 is a potentially new therapeutic target for the treatment of LUAD.

## Supplementary information

Supplementary materials

## Data Availability

For additional details, see the Supplementary material.
