# Supplementary material for: EEF1A2 interacts with HSP90AB1 to promote lung adenocarcinoma metastasis via enhancing TGF-β/SMAD signalling
Source: Br J Cancer. 2021 Jan 21;124(7):1301–11. doi: 10.1038/s41416-020-01250-4 (PMC8007567; doi:10.1038/s41416-020-01250-4)
Supplement: Supplementary file 1 — Supplementary materials [file 41416_2020_1250_MOESM1_ESM.pdf]

Supplementary Material and Method

Subcellular fractionation and western blot analysis

Cells were collected and treated on ice with Radio-Immunoprecipitation Assay Buffer (RIPA, Beyotime Biotechnology, China) supplemented with 10 mM PMSF and protease inhibitor cocktail. The concentration of the lysates was quantified by BCA assay using the BCA^{TM} Protein Assay Kit (Thermo Fisher Scientific, *America*). Subcellular fractionation was performed following the protocol specified for the Nuclear and Cytoplasmic Extraction Reagents (P0027, Beyotime Biotechnology). Equal amount (60 μg) of the samples were separated by 10% Sodium Dodecyl Sulphate-Polyacrylamide Gel Electrophoresis (SDS-PAGE) and transferred to a polyvinylidene difluoride (PVDF) membrane. The blots were blocked with 5% non-fat milk in PBS with Tween 20 for 1.5 h at room temperature and the membranes were incubated with primary antibodies at 4 ℃ overnight. To visualize the protein bands, the membranes were incubated with the corresponding secondary antibody for 1 h at room temperature after washing with PBST and the protein expression was quantified using the BioRad ChemiDoc XRS system.

Cell proliferation assay

LUAD cells were transfected with si-EEF1A2s and corresponding si-CTR and cultured in 6-well plates for 48 h. Then 800 cells/well were seeded in 96-well plates for the CCK8 assay. A 20 μL volume of Vita-Orange Cell Viability Reagent (biotool, China) was added to 200 μL of medium in each well and incubated at 37 ℃ for 2 h. The plates were then read with a microplate reader at 450 nm on a scanning multi-well spectrophotometer (Beckman Coulter, America).

Colony formation assay

Following 48 h of culture post-transfection, the cells were trypsinized and plated at

1000 cells/well in 6-well plates. After 10 days, the cells were gently washed with ice-cold PBS and fixed with 4% paraformaldehyde followed by staining with 0.1% crystal violet. Manual counting was performed using Image Pro Plus 6.0 (Media Cybernetics, America) to calculate the colony forming ability of the treated and control group.

Cell invasion assay

Cell invasion was assayed using Corning Costar Transwell 24-well plates (NY, USA). The transwell chambers were coated with matrigel and placed at 37℃ for 3 h before cell seeding. Cells were seeded at 2×10^4 cells/insert in serum-free culture medium in the upper chamber, and 600 μL of medium containing 20% serum was placed in the lower chamber. The cells that migrated through the matrigel were fixed with 4% paraformaldehyde for 20 min and stained with 0.1% crystal violet for 15 min. Images were captured using Nikon microscope and invasion ability was calculated by counting the stained cells using Image Pro Plus 6.0 (Media Cybernetics, America).

Wound healing assay

A549 and PC9 cells were transfected with Control, si-EEF1A2s or pEnter, pEnter-EEF1A2 and grown to 90% confluency in 6-well plates. A wound was generated in the cell layer in each well using a sterile 10 μL pipette tip and the detached cells were removed by washing with D-Hanks solution. The cells were then incubated in fresh medium containing 2% serum. Images were captured at 0, 24, 48, 72, and 96 h after wounding using a microscope (Olympus corporation, Japan, CKX41SF). Image Pro Plus 6.0 (Media Cybernetics, America) was used to measure the wound area that remained unpopulated by the migrating cells. The migration rate was calculated by dividing the distance migrated in both treated group and control group.

Supplementary Figure legends

Supplementary Figure 1. EEF1A2 increases the proliferation, invasion, and migration ability of LUAD cells *in vitro*

(**A**) The expression of EEF1A2 and EEF1A1 was detected in HBE, A549 and PC9 cells. (**B**) Overexpression of EEF1A2 (pCDH-EEF1A2) in A549 and PC9 cells using a lentiviral vector to stably increase EEF1A2 expression. (**C**) CCK8 assay and (**D**) colony formation assay was performed in EEF1A2 overexpression A549 and PC9 cells (n=3). (**E**) Trans-well assay was performed in EEF1A2 overexpression A549 and PC9 cells. Scale bar=200 μm. Number of invasive cells was shown as mean ± SEM. (**F**) The migration ability was analyzed using a wound-healing assay in EEF1A2 overexpression A549 and PC9 cells (n=3). Scale bar=200 μm. Distance of gap was shown as mean ± SEM. **p* <0.05, ** *p* <0.01, *** *p* <0.001, NS: no statistical significance.

Supplementary Figure 2. EEF1A2 promotes tumor growth and metastasis *in vivo*

(**A**) Western blot analysis of EEF1A2 expression in A549 cells and sh-EEF1A2 cells with luciferase expression. (**B**) Invasive capacities of control (sh-CTR) A549 cells or EEF1A2 knocked down (sh-EEF1A2) A549 cells were monitored by Trans-well assay (n=3). Scale bar=200 μm. Number of invasive cells was shown as mean ± SEM. ****p* < 0.001, NS: no statistical significance. **(C)** Tumor-bearing mice were photographed with inhalational anesthesia, and the arrow indicates the tumor burden, n = 8 mice per group.

Supplementary Figure 3. EEF1A2 interacts with HSP90AB1

(**A**) A549 cells were transfected with Flag-EEF1A2 and the proteins was immunoprecipitated with Flag monoclonal antibody. The EEF1G and XRCC6 were

detected by immunoblotting. (**B**) Flag-EEF1A2 and Myc-HSP90AB1 were transfected into HEK293 cells and the proteins was immunoprecipitated with Flag or Myc antibody separately, Myc-HSP90AB1 or Flag-EEF1A2 was detected by immunoblotting. (**C**) EEF1A2-domainI bind to Myc-HSP90AB1 and HSP90AB1-domainI bind to EEF1A2. (**D**) Confocal images shown position of co-location EEF1A2 (green) and HSP90AB1 (red) after transfection of si-EEF1A2 or si-HSP90AB1 in A549 cells. Images were acquired at 400×. Scale bar = 50 μm.

Supplementary Figure 4. Upregulated HSP90AB1 is associated with poor prognosis of lung adenocarcinoma patients

(**A**) The expression level of HSP90AB1 in HBE, A549 and PC9 cells. (**B**) Western blot analysis of HSP90AB1 expression after si-HSP90AB1 transfected into A549 and PC9 cells. (**C**) Analysis of the HSP90AB1 expression in LUAD (tumor) (n = 45) and normal lung tissues (normal) (n = 65) using the data from GSE19188. (**D**) Over-all survival and relapse-free survival analysis of LUAD patients with low and high expression of HSP90AB1 using Kaplan-Meier curve analysis. *p <0.05, ** p <0.01, *** p <0.001, NS: no statistical significance.

Supplementary Figure 1

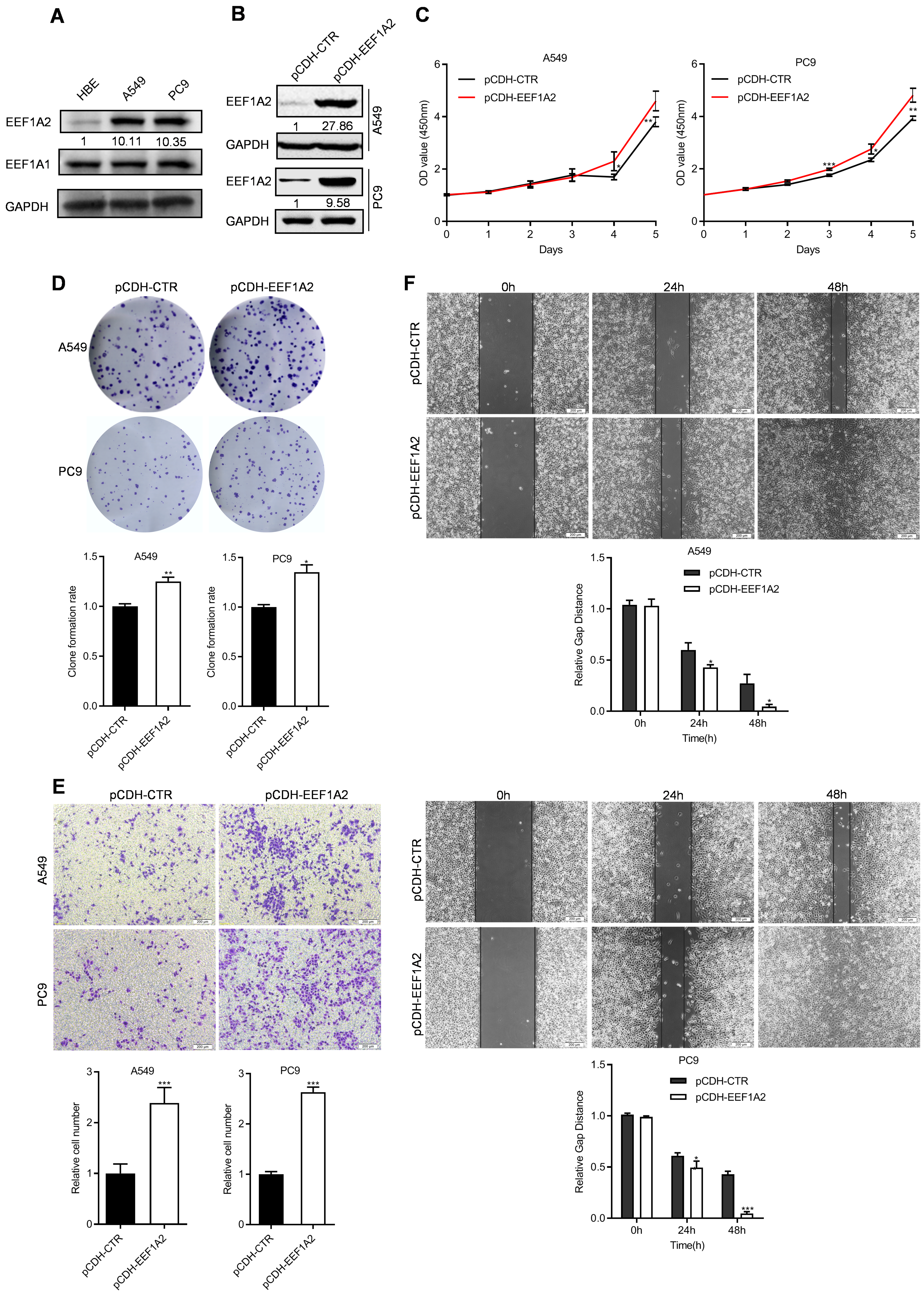

Supplementary Figure 2

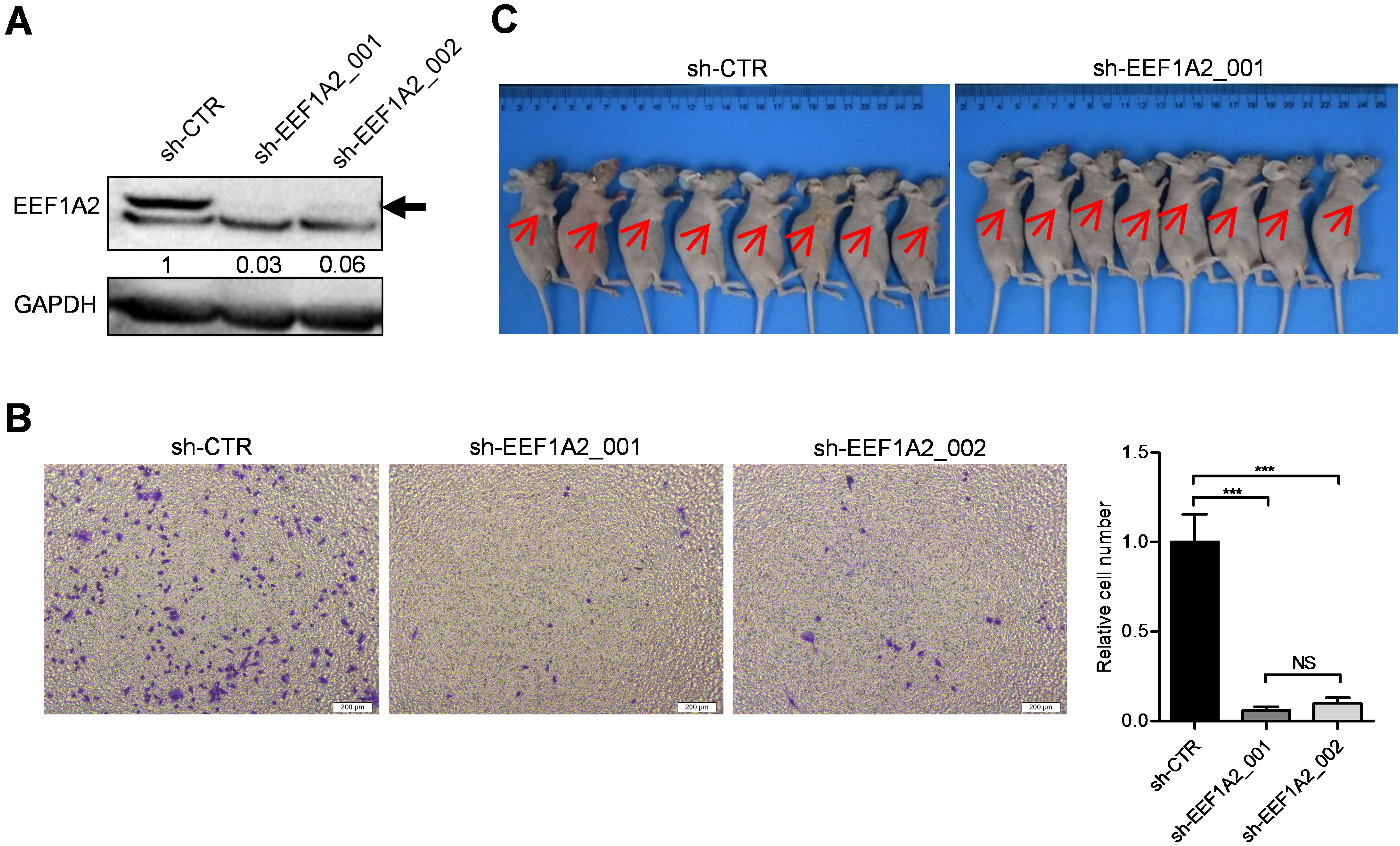

Supplementary Figure 3

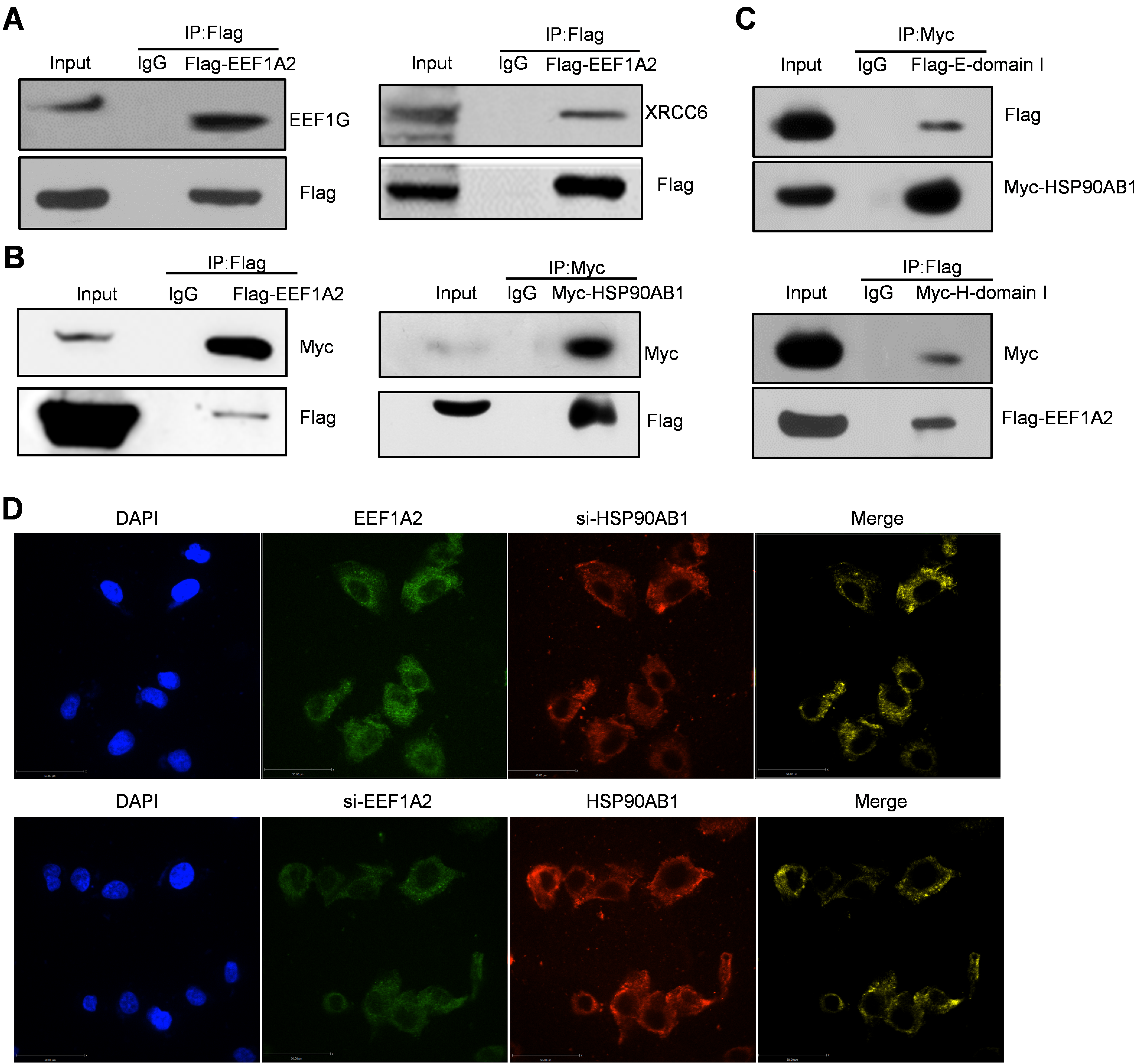

Supplementary Figure 4

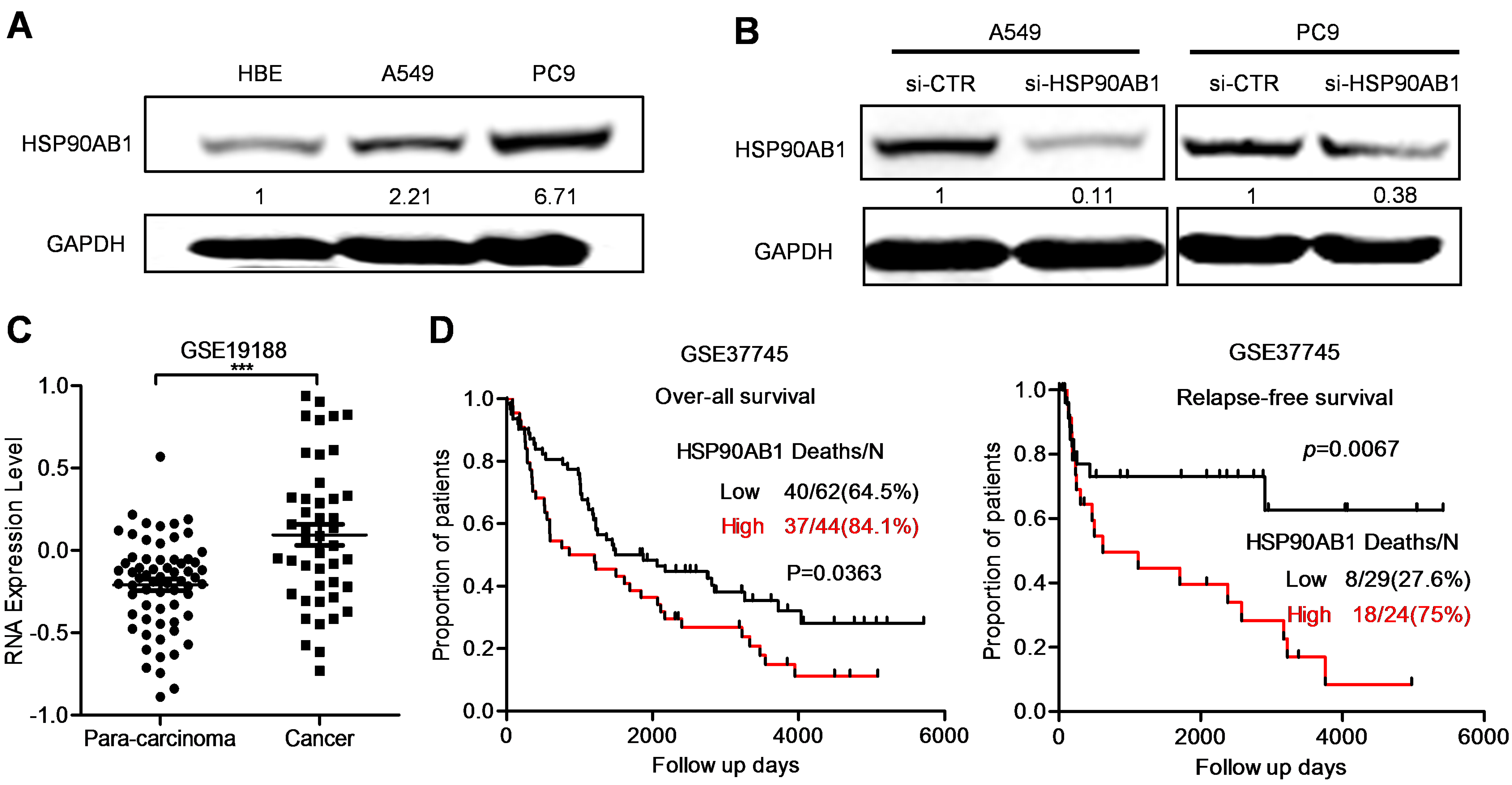

Supplementary Table1: Clinicopathological data on 160 paraffin-embedded LUAD biopsies.

Patient No.	Gender	Age	T stage	N stage	M stage	Pathological stage	Histopathological type
Pat 001	F	58	T2b	N0	M0	Ⅲ	carcinoma
							para-carcinoma
Pat 002	F	63	T2a	Nx	M0	Ⅱ-Ⅲ	carcinoma
							para-carcinoma
Pat 003	M	67	T2a	N2	M0	Ⅱ	carcinoma
							para-carcinoma
Pat 004	F	59	T1b	Nx	M0	Ⅱ	carcinoma
							para-carcinoma
Pat 005	F	60	T1b	Nx	M0	Ⅱ	carcinoma
							para-carcinoma
Pat 006	M	71	T3	N1	M0	Ⅱ	carcinoma
							para-carcinoma
Pat 007	F	60	T3	N3	M0	Ⅱ	carcinoma
							para-carcinoma
Pat 008	M	61	T2b	N3	M0	Ⅲ	carcinoma
							para-carcinoma
Pat 009	F	58	T3	N0	M0	Ⅰ-Ⅱ	carcinoma
							para-carcinoma
Pat 010	M	66	T2b	N0	M0	Ⅲ	carcinoma
							para-carcinoma
Pat 011	M	60	T3	N1	M0	Ⅱ	carcinoma
							para-carcinoma
Pat 012	M	63	T3	N2	M0	Ⅱ	carcinoma
							para-carcinoma
Pat 013	F	65	T2a	N1	M0	Ⅱ	carcinoma
							para-carcinoma
Pat 014	M	58	T2a	N1	M0	Ⅱ	carcinoma
							para-carcinoma
Pat 015	M	61	T2a	N0	M0	Ⅱ-Ⅲ	carcinoma
							para-carcinoma

Pat 016	F	81	T2a	N1	M0	II	carcinoma<br>para-carcinoma
Pat 017	F	60	T1b	N0	M0	II	carcinoma<br>para-carcinoma
Pat 018	F		T1a	Nx	M0	II	carcinoma<br>para-carcinoma
Pat 019	M	65	T2a	N2	M0	II	carcinoma<br>para-carcinoma
Pat 020	M	53	T2a	N0	M0	III	carcinoma<br>para-carcinoma
Pat 021	F	50	T1b	Nx	M0	II	carcinoma<br>para-carcinoma
Pat 022	M	64	T1b	N0	M0	II	carcinoma<br>para-carcinoma
Pat 023	M	64	T2a	N1	M0	II	carcinoma<br>para-carcinoma
Pat 024	M	53	T2a	N0	M0	III	carcinoma<br>para-carcinoma
Pat 025	F		T1b	N0	M0	I	carcinoma<br>para-carcinoma
Pat 026	F	73	T4	N2	M0	II	carcinoma<br>para-carcinoma
Pat 027	M	52	T2a	N2	M0	II	carcinoma<br>para-carcinoma
Pat 028	M	37	T2a	N0	M0	II	carcinoma<br>para-carcinoma
Pat 029	F	73	T2b	Nx	M0	II	carcinoma<br>para-carcinoma
Pat 030	M	44	T3	Nx	M0	II	carcinoma<br>para-carcinoma
Pat 031	M	61	T1a	N0	M0	I	carcinoma<br>para-carcinoma
Pat 032	M	55	T2b	N2	M0	II	carcinoma<br>para-carcinoma

Pat 033	F	50	T1a	N0	M0	II	carcinoma<br>para-carcinoma
Pat 034	M	68	T1b	N0	M0	II	carcinoma<br>para-carcinoma
Pat 035	M	78	T1b	N0	M0	II	carcinoma<br>para-carcinoma
Pat 036	F	60	T2b	N0	M0	II	carcinoma<br>para-carcinoma
Pat 037	F	54	T3	N1	M0	II	carcinoma<br>para-carcinoma
Pat 038	F	48	T3	N0	M0	II	carcinoma<br>para-carcinoma
Pat 039	M	48	T2a	N2	M0	II	carcinoma<br>para-carcinoma
Pat 040	M	40	T1b	Nx	M0	II	carcinoma<br>para-carcinoma
Pat 041	M	58	T2a	N0	M0	III	carcinoma<br>para-carcinoma
Pat 042	F	56	T4	N2	M0	II	carcinoma<br>para-carcinoma
Pat 043	F	53	T2a	N0	M0	I	carcinoma<br>para-carcinoma
Pat 044	F	62	T1b	Nx	M0	II	carcinoma<br>para-carcinoma
Pat 045	M	57	T2a	Nx	M0	II	carcinoma<br>para-carcinoma
Pat 046	M	52	T2a	N0	M0	II	carcinoma<br>para-carcinoma
Pat 047	F	40	T2a	N0	M0	I - II	carcinoma<br>para-carcinoma
Pat 048	M	61	T2b	N0	M0	I - II	carcinoma<br>para-carcinoma
Pat 049	F	65	T2a	N0	M0	II	carcinoma<br>para-carcinoma

Pat 050	M	41	T1a	N0	M0	II	carcinoma
							para-carcinoma
Pat 051	F	67	T1b	Nx	M0	II	carcinoma
							para-carcinoma
Pat 052	M	60	T2a	N0	M0	III	carcinoma
							para-carcinoma
Pat 053	F	57	T1b	N0	M0	II	carcinoma
							para-carcinoma
Pat 054	F	56	T2a	N0	M0	II	carcinoma
							para-carcinoma
Pat 055	M	65	T3	N2	M0	III	carcinoma
							para-carcinoma
Pat 056	M	65	T3	N0	M0	III	carcinoma
							para-carcinoma
Pat 057	F	77	T3	N3	M0	II	carcinoma
							para-carcinoma
Pat 058	F	67	T1a	N0	M0	II	carcinoma
							para-carcinoma
Pat 059	M	67	T1b	N0	M0	II	carcinoma
							para-carcinoma
Pat 060	M	67	T2b	Nx	M0	II	carcinoma
							para-carcinoma
Pat 061	M	50	T2a	N0	M0	III	carcinoma
							para-carcinoma
Pat 062	M	50	T2	N0	M0	II	carcinoma
							para-carcinoma
Pat 063	F	66	T2a	N0	M0	I	carcinoma
							para-carcinoma
Pat 064	F	68	T2a	N0	M0	I	carcinoma
							para-carcinoma
Pat 065	F	68	T1b	N0	M0	II	carcinoma
							para-carcinoma
Pat 066	F	63	T2a	N0	M0	II -III	carcinoma
							para-carcinoma

Pat 067	M	60	T2a	N0	M0	III	carcinoma
							para-carcinoma
Pat 068	M	60	T1b	N1	M0	II-III	carcinoma
							para-carcinoma
Pat 069	F	60	T2a	N1	M0	III	carcinoma
							para-carcinoma
Pat 070	F	75	T2a	N2	M0	II	carcinoma
							para-carcinoma
Pat 071	M	52	T2a	N2	M0	II-III	carcinoma
							para-carcinoma
Pat 072	F	50	T2a	N2	M0	II-III	carcinoma
							para-carcinoma
Pat 073	M	56	T2a	N3	M0	II	carcinoma
							para-carcinoma
Pat 074	M	45	T4		M0	II	carcinoma
							para-carcinoma
Pat 075	F				M1b	II-III	carcinoma
							para-carcinoma
Pat 076	F	65	T2a	N2	M1b	II	carcinoma
							para-carcinoma
Pat 077	M	69	T3	N2	M1b	II-III	carcinoma
							para-carcinoma
Pat 078	M	57	T2a	N0	M1b	II	carcinoma
							para-carcinoma
Pat 079	F	45			M1b		Metastatic lung adenocarcinoma
Pat 080	F	58			M1b		Metastatic lung adenocarcinoma
Pat 081	F	65			M1b		Metastatic lung adenocarcinoma
Pat 082	M	65			M1b		Metastatic lung adenocarcinoma

Supplementary Table2: Information on antibodies used in this study.

Antibody	Company	Catalog #	Species	Dilution
EEF1A2	ABclonal	A2473	Rabbit	1:1000
GAPDH	ABclonal	AC001	Rabbit	1:1000
Lamin A/C	Proteintech	10298-1-AP	Rabbit	1:5000
HRP-conjugated Affinipure Goat Anti-Mouse IgG(H+L)	Proteintech	SA00001-1	Mouse	1:5000
HRP-conjugated Affinipure Goat Anti-Rabbit IgG(H+L)	Proteintech	SA00001-2	Rabbit	1:5000
Alexa Fluor 594-conjugated Goat anti Rabbit	Proteintech	SA00006-4	Rabbit	1:2000
Alexa Fluor 488-conjugated Goat anti Mouse	Proteintech	SA00006-1	Mouse	1:2000
Flag	SIGMA	F1804	Mouse	1:500
Myc	CUSABIO	16286-1-AP	Rabbit	1:100
XRCC6	CUSABIO	PA01617A0Rb	Rabbit	1:2000
Anti-rabbit IgG	Cell Signaling Technology	7074	Goat	1:2000
N-Cadherin	ABclonal	A0433	Rabbit	1:1000
SNAI2(Slug)	Affinity	AF4002	Rabbit	1:1000
Pierce^{TM} SMAD3 Antibody(E.980.9)	Thermo SCIENTIFIC	QK2117221H	Rabbit	1:1000
Pierce^{TM} phospho-SMAD3 pSer423+425 Antibody(S.434.0	Thermo SCIENTIFIC	QK21173441F	Rabbit	1:1000
TGFBR I (TβR I)	Affinity	AF5347	Rabbit	1:1000
TGF beta Receptor II (TβR II)	Abways	F058501	Rabbit	1:1000
EEF1A1	ABclonal	AF17857	Rabbit	1:1000
HSP90AB1	Affinity	BF0215	Mouse	1:1000

Supplementary Table3: The sequences of siRNAs and shRNAs used in this study.

	sequences
si-EEF1A2_001	5'-CACCGCGACTTCATCAAGA-3'
si-EEF1A2_002	5'-CCGTAGGCGTCATCAAGAA-3'
si-HSP90AB1	5’-GGAAGAGAAAGGUGAGAAA-3’
sh-EEF1A2_001	5'-GATCCATCAAGAAGATCGGCTACAACTCGAGTTGTAGCCGATCTTCTTGATGTTTTTTG-3'
sh-EEF1A2_002	5'-GATCACCATTGAGAAGTTCGAGAAGCTCGAGCTTCTCGAACTTCTCAATGGTTTTTTG-3'

Supplementary Table 4: Identification of 708 proteins using LC-MS/MS in A549 cell.

Accession	Gene Symbol	Score	Coverage	# Proteins	# Unique Peptides	# Peptides	# PSMs	Area	# AAs
Q05639	EEF1A2	3583.00	72.57	1	10	23	1166	7.405E9	463
P68104	EEF1A1	2519.61	71.21	6	8	21	800	6.831E9	462
P26641	EEF1G	1781.65	76.89	1	29	29	676	6.078E9	437
E9PRY8	EEF1D	1152.82	37.88	27	12	26	414	4.233E9	697
P26640	VARS	1055.81	44.46	7	52	52	409	8.273E8	1264
Q86UP2	KTN1	834.54	58.59	10	73	73	339	2.033E8	1357
P60709	ACTB	627.72	60.80	25	8	16	242	1.640E9	375
E9PL71	EEF1D	611.38	73.26	5	1	12	218	3.761E9	187
O14744	PRMT5	610.27	56.20	14	27	27	209	5.455E8	637
P21333	FLNA	476.88	44.69	8	75	79	188	4.670E7	2647
E9PK06	EEF1D	470.58	64.75	5	1	8	181	1.415E9	139
P52732	KIF11	431.39	50.09	1	50	50	170	1.617E8	1056
P24534	EEF1B2	425.96	79.11	4	9	13	127	3.025E9	225
A0A0A0MRV0	RRBP1	399.37	34.61	10	35	36	129	6.373E7	1410
P68371	TUBB4B	391.15	72.36	22	4	22	139	2.146E8	445
P68032	ACTC1	372.06	26.53	18	1	9	164	1.238E9	377
P07437	TUBB	362.30	69.14	18	4	21	131	2.202E8	444
Q13813	SPTAN1	330.18	28.20	7	52	52	129	1.188E7	2472
Q01082	SPTBN1	314.70	34.56	5	57	57	119	1.475E7	2364
A0A0C4DGB6	ALB	294.05	13.41	8	8	8	113	3.173E8	604
O60256	PRPSAP2	293.43	56.37	14	14	16	98	7.666E7	369
P11142	HSPA8	273.44	41.64	14	8	21	112	8.770E7	646
O75369	FLNB	254.53	19.52	5	34	38	96	2.512E7	2602
Q14558	PRPSAP1	242.00	51.69	6	11	13	88	8.066E7	356
P62701	RPS4X	222.40	50.57	4	14	14	89	9.130E7	263
Q9BQE3	TUBA1C	214.15	51.22	13	5	17	90	3.354E8	449
Q7Z2W4	ZC3HAV1	184.08	21.84	3	15	15	71	1.881E7	902
P11908	PRPS2	178.31	48.74	4	5	12	67	1.518E8	318
P39023	RPL3	174.57	40.45	7	16	16	63	5.838E7	403
P68366	TUBA4A	173.18	54.69	11	6	18	70	3.029E8	448
P0DMV8	HSPA1A	172.02	31.36	4	14	16	69	7.660E7	641
E9PK54	HSPA8	169.79	86.34	5	1	13	65	8.472E7	183
Q8IY21	DDX60	169.43	22.02	5	31	31	68	1.993E7	1712

P08670	VIM	164.52	51.93	17	22	23	69	7.586E7	466
P11021	HSPA5	162.88	38.07	1	17	19	58	6.926E7	654
Q9BQA1	WDR77	159.15	57.60	2	10	10	64	3.392E8	342
Q07065	CKAP4	155.96	45.18	6	21	21	54	2.761E7	602
P60891	PRPS1	154.21	57.23	4	7	14	55	1.498E8	318
O43707	ACTN4	150.97	40.94	10	18	27	70	3.093E7	911
P38646	HSPA9	148.90	33.58	5	18	18	53	2.016E7	679
A0A0D9SFB3	DDX3X	146.40	35.16	15	19	20	58	2.783E7	640
P36578	RPL4	145.78	40.98	4	16	16	66	7.626E7	427
Q01813	PFKP	140.83	22.32	8	11	13	51	1.549E7	784
P07355	ANXA2	137.21	46.90	24	16	16	50	7.261E7	339
Q9Y6Y0	IVNS1ABP	133.37	38.94	3	18	18	58	1.178E8	642
O75688	PPM1B	131.30	30.48	7	11	11	52	2.093E7	479
P42704	LRPPRC	128.29	19.58	4	20	20	46	1.394E7	1394
P12814	ACTN1	127.19	34.64	14	14	23	52	3.093E7	892
P80723	BASP1	124.77	67.40	2	10	10	40	1.429E7	227
P62906	RPL10A	120.22	29.95	1	7	7	53	4.030E7	217
P46940	IQGAP1	119.54	14.79	5	17	17	45	1.116E7	1657
P19338	NCL	118.75	19.58	6	12	12	51	2.954E7	710
Q00839	HNRNPU	116.82	23.15	2	15	15	35	6.335E7	825
P23396	RPS3	116.57	63.37	13	13	13	41	8.196E7	243
A0A096LNZ9	ISG15	115.25	42.66	3	6	6	49	7.443E7	143
P08238	HSP90AB1	114.94	26.38	9	8	15	48	3.049E7	724
Q14166	TTLL12	114.56	35.56	2	17	17	50	2.571E7	644
Q14258	TRIM25	114.37	18.57	1	10	10	51	6.004E6	630
Q13283	G3BP1	107.81	39.70	7	12	12	48	2.017E7	466
P20591	MX1	103.38	26.13	8	11	12	42	9.034E6	662
P46777	RPL5	102.34	38.05	2	9	9	38	3.300E7	297
Q9BUF5	TUBB6	96.79	28.25	10	3	8	33	2.057E8	446
P67809	YBX1	96.66	41.36	4	5	8	30	2.669E7	324
P04792	HSPB1	94.53	49.76	3	8	8	25	5.273E7	205
J3QR09	RPL19	93.82	22.28	4	4	4	43	4.325E7	193
Q02878	RPL6	92.96	43.75	7	14	14	44	5.164E7	288
P35637	FUS	91.01	17.68	4	6	6	32	2.524E7	526
Q06830	PRDX1	90.84	56.78	5	10	11	36	5.780E7	199
P62280	RPS11	87.35	41.14	3	8	8	37	3.471E7	158

P61247	RPS3A	87.10	48.11	12	12	12	40	6.228E7	264
E9PCY7	HNRNPH1	84.26	21.45	24	5	7	35	3.068E7	429
J3KTA4	DDX5	83.30	27.04	9	13	17	42	3.572E7	614
H3BLV0	CD55	82.49	19.63	5	6	6	35	3.760E7	326
P62241	RPS8	81.94	51.92	2	11	11	31	1.303E8	208
Q86YZ3	HRNR	81.15	7.12	1	6	6	30	5.208E6	2850
E9PMS6	LMO7	80.66	18.35	15	16	16	31	7.631E6	1275
P63244	RACK1	80.56	52.68	22	11	11	27	2.483E7	317
P17858	PFKL	78.89	13.59	1	5	8	27	7.784E6	780
P15880	RPS2	78.22	43.69	9	11	11	35	8.221E7	293
Q08211	DHX9	78.14	12.99	1	13	13	29	1.245E7	1270
Q9UII4	HERC5	77.46	17.58	2	14	14	32	1.035E7	1024
P61978	HNRNPK	77.23	31.53	4	10	10	27	3.071E7	463
Q14764	MVP	76.88	17.36	6	11	11	35	9.170E6	893
P08708	RPS17	76.44	57.04	5	8	8	29	3.167E7	135
Q9Y5B9	SUPT16H	76.30	10.89	1	10	10	33	9.405E6	1047
P07814	EPRS	73.34	13.56	3	16	16	33	5.831E6	1512
P61313	RPL15	73.19	39.22	5	7	7	30	4.601E7	204
O43143	DHX15	72.38	23.52	5	16	16	30	1.025E7	795
Q562R1	ACTBL2	70.54	15.69	6	1	5	52	1.532E9	376
P06576	ATP5B	68.50	23.06	5	8	8	23	7.966E6	529
P35579	MYH9	68.45	9.85	6	14	14	27	5.955E6	1960
Q15208	STK38	68.12	30.11	4	9	9	28	3.015E7	465
A8MUD9	RPL7	67.89	36.06	4	8	8	25	5.312E7	208
H0YHA7	RPL18	67.27	43.71	7	7	7	24	1.158E8	167
E7EQR4	EZR	66.77	12.46	8	4	7	29	5.558E6	586
F8W6I7	HNRNPA1	66.41	36.16	8	9	10	31	2.558E7	307
P26038	MSN	66.13	9.71	5	3	6	31	5.405E6	577
A0A087X0X3	HNRNPM	65.63	24.38	10	14	14	29	8.918E6	730
B4DKY1	CARS	64.62	17.86	4	9	10	27	1.138E7	739
P61254	RPL26	63.95	38.62	10	2	7	26	4.192E7	145
P32969	RPL9	63.92	50.00	5	6	6	21	4.711E7	192
P05388	RPLP0	62.27	41.64	13	9	9	21	1.050E8	317
Q07955	SRSF1	62.10	44.35	5	10	10	27	1.319E7	248
P62249	RPS16	61.99	43.84	7	7	7	23	5.184E7	146
P14868	DARS	61.35	21.76	6	9	9	27	8.072E6	501

B4DNK4	PKM	61.32	27.13	12	10	10	20	1.373E7	457
A0A087WXM6	RPL17	60.91	45.56	12	7	7	31	7.139E7	169
Q16643	DBN1	60.52	14.95	4	6	6	19	2.251E7	649
Q15646	OASL	59.76	23.93	2	10	10	24	2.122E7	514
P25205	MCM3	59.70	17.57	3	11	11	25	3.970E6	808
D3YTB1	RPL32	59.37	30.08	3	5	5	23	1.724E7	133
E7EPB3	RPL14	58.97	36.29	2	4	4	23	3.054E7	124
P10809	HSPD1	58.14	18.15	6	8	8	21	7.408E6	573
K7EM56	RPS15	57.31	40.18	8	4	4	20	1.550E7	112
P04406	GAPDH	57.05	33.73	3	8	8	21	2.542E7	335
P06748	NPM1	57.01	27.89	3	6	6	19	2.520E7	294
Q92841	DDX17	56.49	14.68	3	5	9	24	3.024E7	729
J3KR24	IARS	55.66	11.98	5	10	10	19	6.948E6	1152
P69905	HBA1	55.58	21.83	2	2	2	28	1.335E7	142
P06733	ENO1	54.53	16.82	7	6	6	21	1.168E7	434
P47897	QARS	54.15	10.32	17	7	7	23	5.236E6	775
F8VZJ2	NACA	53.65	40.44	9	4	4	16	2.082E7	136
A0A087WTT1	PABPC1	53.62	23.95	24	6	10	22	1.176E7	522
P62424	RPL7A	53.56	31.95	3	9	9	24	8.317E7	266
P21291	CSRP1	53.44	47.15	4	6	6	17	3.012E7	193
X1WI28	RPL10	53.09	44.00	9	1	7	26	4.053E7	200
P05141	SLC25A5	53.01	25.84	2	3	7	21	5.856E7	298
P52597	HNRNPF	52.74	16.39	2	3	5	20	2.325E7	415
P54136	RARS	52.10	13.03	3	7	7	17	8.762E6	660
A0A087X1H5	ACAP2	51.81	17.76	6	9	9	19	7.938E6	777
Q9P258	RCC2	51.71	21.84	1	8	8	20	8.182E6	522
P07900	HSP90AA1	51.66	16.53	9	4	10	22	2.927E7	732
K7ENG2	U2AF2	51.15	28.01	2	5	5	15	9.325E6	307
P40429	RPL13A	50.62	38.42	6	9	9	25	6.724E7	203
P98175	RBM10	50.26	14.30	2	10	10	18	9.790E6	930
F8W7C6	RPL10	48.56	48.47	6	1	7	25	3.721E7	163
P30050	RPL12	48.41	49.09	1	5	5	15	5.169E7	165
E7EVX8	PRPF31	48.22	23.33	5	8	8	20	1.155E7	493
E9PMI6	CLNS1A	47.76	26.35	5	3	3	18	3.011E7	167
B1AHC9	XRCC6	47.65	23.26	4	10	10	18	9.533E6	559
Q15233	NONO	46.76	20.17	5	7	7	20	1.158E7	471

Q9P2J5	LARS	46.60	8.33	2	7	7	18	4.757E6	1176
O95831	AIFM1	46.55	15.99	2	8	8	18	3.370E6	613
P62269	RPS18	46.33	47.37	4	9	9	20	5.484E7	152
O60506	SYNCRIP	45.59	17.66	5	9	9	20	1.294E7	623
P16989	YBX3	44.76	23.92	3	3	6	16	1.240E7	372
P41091	EIF2S3	44.54	18.64	4	6	6	20	1.063E7	472
P26373	RPL13	43.97	29.38	3	6	6	17	8.874E7	211
P22626	HNRNPA2B1	43.94	26.91	2	7	8	23	1.545E7	353
P13639	EEF2	43.52	16.08	3	10	11	22	7.597E6	858
B4DUR8	CCT3	43.51	19.20	8	7	8	17	6.124E6	500
P19525	EIF2AK2	43.37	12.16	3	5	5	16	6.332E6	551
A0A0A0MRA5	HNRNPUL1	42.62	5.09	10	3	3	16	3.656E6	766
Q96HC4	PDLIM5	42.39	13.93	4	6	6	17	3.526E6	596
Q14444	CAPRIN1	42.20	23.13	3	8	8	14	1.314E7	709
Q9NXV2	KCTD5	42.12	23.08	2	4	4	15	8.987E6	234
P46778	RPL21	42.01	27.50	3	3	3	14	4.586E7	160
P62851	RPS25	41.68	29.60	1	5	5	19	8.388E7	125
E5RJU9	MTDH	41.15	10.84	3	4	4	19	4.405E6	526
F8VVM2	SLC25A3	40.73	17.59	2	5	5	15	1.202E7	324
C9J9K3	RPSA	40.57	21.67	4	3	3	13	7.654E6	263
P50990	CCT8	40.42	18.25	3	9	9	20	5.898E6	548
Q9BRS2	RIOK1	40.33	14.44	2	7	7	20	8.451E6	568
C9JAK5	ARF4	38.93	33.33	5	3	4	15	1.221E7	153
P52701	MSH6	38.93	6.62	3	7	7	18	3.188E6	1360
P46781	RPS9	38.92	40.72	5	12	12	19	6.349E7	194
O00622	CYR61	38.51	14.70	1	5	5	18	7.526E6	381
C9JXB8	RPL24	38.25	31.40	3	4	4	15	4.213E7	121
P62266	RPS23	38.19	16.08	2	2	2	13	2.909E7	143
Q92522	H1FX	37.07	17.37	1	3	3	12	7.114E6	213
A0A0A0MSQ0	PLS3	37.03	18.15	7	9	9	17	4.673E6	617
P02545	LMNA	36.75	17.02	5	8	8	16	3.633E6	664
P12236	SLC25A6	36.59	21.14	4	2	6	16	5.828E7	298
O75396	SEC22B	35.95	16.74	2	3	3	12	2.816E6	215
P62917	RPL8	35.44	19.07	5	5	5	14	4.223E7	257
B1ANR0	PABPC4	35.35	13.50	8	2	6	14	1.176E7	615
A6NHL2	TUBAL3	35.05	10.31	1	1	5	17	2.190E7	446

Q9UNX3	RPL26L1	35.05	32.41	4	1	6	15	3.631E7	145
P29728	OAS2	35.04	9.60	2	5	5	13	8.426E6	719
P62753	RPS6	35.03	21.29	3	5	5	12	8.970E7	249
P49411	TUFM	34.88	17.70	1	8	8	15	2.278E7	452
P07477	PRSS1	34.69	15.38	13	3	3	25	4.701E8	247
P51991	HNRNPA3	34.13	24.34	2	6	7	13	6.831E6	378
P62263	RPS14	33.64	36.42	3	5	5	11	7.863E7	151
O95573	ACSL3	33.64	8.19	2	4	4	12	5.683E6	720
Q8NE71	ABCF1	33.63	9.35	5	6	6	11	4.841E6	845
H3BRU6	PCBP2	33.59	11.96	13	2	3	16	4.786E6	301
P20592	MX2	33.55	7.13	2	2	3	9	4.701E6	715
E7ETK0	RPS24	33.50	20.61	3	3	3	14	4.741E7	131
P62750	RPL23A	33.41	33.33	6	6	6	18	8.346E7	156
P05387	RPLP2	32.65	69.57	2	5	5	10	1.540E7	115
J3QLE5	SNRPN	32.60	13.61	5	2	2	12	1.195E7	169
Q6NZI2	PTRF	32.34	9.49	1	3	3	11	4.961E6	390
Q15046	KARS	32.29	10.05	3	5	5	12	5.058E6	597
P27816	MAP4	32.06	7.99	6	7	7	15	4.928E6	1152
C9JQV0	C7orf50	31.66	35.94	4	4	4	11	3.600E6	192
P53999	SUB1	31.60	51.97	3	6	6	13	4.125E7	127
Q08945	SSRP1	31.50	13.40	3	7	7	14	8.364E6	709
Q8NC51	SERBP1	31.49	12.99	1	4	4	12	1.603E7	408
Q9Y6K5	OAS3	31.15	7.27	3	7	7	14	5.741E6	1087
O75533	SF3B1	30.72	6.44	2	5	5	10	1.865E6	1304
Q13151	HNRNPA0	30.69	18.36	1	3	4	11	6.952E6	305
P04843	RPN1	30.41	15.65	3	7	7	13	5.076E6	607
P08237	PFKM	29.82	6.92	2	3	5	11	4.163E6	780
Q02543	RPL18A	29.69	38.07	5	7	7	18	3.478E7	176
H0YMZ1	PSMA4	29.52	15.45	10	3	3	13	4.737E6	220
P56192	MARS	29.31	7.33	6	5	5	11	3.304E6	900
Q9Y3Z3	SAMHD1	29.10	10.54	1	5	5	14	3.064E6	626
Q99613	EIF3C	29.03	7.78	3	4	4	9	3.213E6	913
P14923	JUP	28.78	8.19	7	5	5	13	4.061E6	745
M0R0F0	RPS5	28.51	31.50	4	5	5	12	2.314E7	200
Q08380	LGALS3BP	28.43	7.86	10	4	4	12	3.183E6	585
O95373	IPO7	28.38	5.97	1	5	5	10	4.140E6	1038

B1AK87	CAPZB	28.34	22.31	4	5	5	13	2.697E6	260
A0A087WUK2	HNRNPDL	28.23	14.88	3	3	5	12	8.109E6	363
Q96AG4	LRRC59	27.98	20.52	2	5	5	10	2.009E7	307
Q5T9A4	ATAD3B	27.94	9.57	4	6	6	15	4.333E6	648
Q5VU61	TPM3	27.90	10.31	15	1	2	9	3.159E6	223
P16403	HIST1H1C	27.55	10.80	5	2	2	14	5.663E6	213
P52907	CAPZA1	27.43	27.97	5	5	5	13	4.961E6	286
E5RIU6	CDK1	27.32	29.63	10	5	5	12	4.032E6	189
P04075	ALDOA	26.96	17.58	9	4	4	9	3.942E6	364
C9J3L8	SSR1	26.70	12.83	5	3	3	11	4.278E6	265
H0YFA4	CRIP2	26.19	25.00	2	2	2	7	1.056E7	192
O14879	IFIT3	26.18	8.98	1	4	4	13	1.790E6	490
D6RF44	HNRNPD	26.06	39.64	8	2	4	10	1.049E7	111
C9J9W2	LASP1	26.05	30.12	4	5	5	11	8.260E6	166
O00425	IGF2BP3	25.92	13.82	3	6	6	11	3.483E6	579
E7ENZ3	CCT5	25.89	16.67	4	6	7	13	3.927E6	486
Q12797	ASPH	25.78	16.89	7	8	8	10	1.391E7	758
P04899	GNAI2	25.72	11.83	1	3	3	9	3.570E6	355
Q12904	AIMP1	25.46	15.71	1	3	3	11	3.266E6	312
Q13263	TRIM28	25.08	7.90	4	5	5	10	9.051E6	835
P37108	SRP14	24.63	38.24	3	3	3	10	1.706E7	136
P31689	DNAJA1	24.57	6.55	1	2	2	9	7.329E6	397
P63104	YWHAZ	24.57	9.80	10	1	2	7	5.617E6	245
P12004	PCNA	24.49	15.33	1	3	3	17	3.283E6	261
E9PLJ3	CFL1	24.49	49.37	8	3	3	5	1.557E7	79
Q9Y3U8	RPL36	24.42	36.19	5	5	5	14	6.906E6	105
E9PLL6	RPL27A	24.37	37.96	4	4	4	13	6.134E7	108
Q9NZ01	TECR	24.34	10.06	3	3	3	10	3.272E6	308
Q9Y3F4	STRAP	24.33	25.14	2	6	6	10	2.446E6	350
A0A0J9YYL3	PUF60	24.33	5.54	12	3	3	15	2.050E6	505
Q6PKG0	LARP1	24.32	7.57	6	6	6	10	4.432E6	1096
P39019	RPS19	24.08	47.59	6	8	8	12	1.352E7	145
F5H6Q2	UBC	23.33	33.61	22	2	2	7	1.077E7	122
P62829	RPL23	23.24	36.43	5	4	4	13	2.245E7	140
P84103	SRSF3	22.92	36.59	2	5	6	14	2.901E7	164
P30041	PRDX6	22.91	17.41	1	4	4	14	4.252E6	224

E5RI99	RPL30	22.84	48.25	5	5	5	12	4.393E6	114
P62277	RPS13	22.56	38.41	3	6	6	10	4.781E7	151
P61204	ARF3	22.50	28.18	7	3	4	8	4.820E6	181
P61353	RPL27	22.48	30.15	3	3	3	11	4.197E7	136
Q15365	PCBP1	22.34	7.02	2	1	2	10	4.842E6	356
P47914	RPL29	22.26	14.47	1	2	2	9	5.121E7	159
P23284	PPIB	22.05	11.11	1	2	2	8	6.536E6	216
H0YL52	TPM1	21.96	12.45	20	2	3	8	3.325E6	265
Q9Y265	RUVBL1	21.89	7.89	2	3	3	8	4.643E6	456
Q9Y657	SPIN1	21.71	20.23	2	4	4	9	4.635E6	262
Q02809	PLOD1	21.60	6.74	3	3	3	7	3.428E6	727
O60814	HIST1H2BK	21.46	11.90	15	1	1	6	8.823E6	126
P05386	RPLP1	21.24	51.75	1	2	2	6	3.982E7	114
P05198	EIF2S1	21.21	25.71	3	7	7	13	8.021E6	315
Q8WUT4	LRRN4	21.13	2.70	1	1	1	7	2.690E9	740
Q9P035	HACD3	21.05	9.67	5	2	2	8	9.406E6	362
Q12906	ILF3	20.89	6.15	9	5	5	10	4.722E6	894
P32119	PRDX2	20.81	18.18	2	2	3	9	1.829E7	198
B5MCP9	RPS7	20.55	22.99	2	4	4	7	3.400E7	187
Q9NZN4	EHD2	20.47	6.45	1	3	3	9	1.965E6	543
P62316	SNRPD2	20.44	41.53	2	4	4	9	3.992E6	118
Q9NR12	PDLIM7	20.21	11.82	5	4	4	10	5.009E6	457
P51571	SSR4	20.21	18.50	2	2	2	8	7.637E6	173
P35249	RFC4	20.00	14.33	6	4	4	7	1.184E6	363
Q9UG63	ABCF2	19.95	8.03	3	5	5	10	4.865E6	623
H0YFC6	RAN	19.92	29.13	5	3	3	10	4.714E6	103
P62854	RPS26	19.87	33.91	2	3	3	9	2.935E7	115
A6NLN1	PTBP1	19.72	6.83	12	4	4	9	5.536E6	527
P08754	GNAI3	19.70	4.24	1	1	1	7	3.371E6	354
Q9UN86	G3BP2	19.66	4.98	1	2	2	7	2.273E6	482
E9PMD7	PPP1CA	19.53	16.21	14	3	3	6	7.386E5	253
B1AHB1	MCM5	19.51	8.10	3	4	4	10	6.458E6	691
A0A087X2G1	DDX1	19.41	8.35	3	4	4	8	3.339E6	659
Q15427	SF3B4	19.23	3.30	1	1	1	6	6.285E7	424
Q5F1R6	DNAJC21	19.01	8.10	1	3	3	8	2.315E6	531
A0A087X253	AP2B1	18.83	7.56	7	5	5	8	4.278E6	913

Q9ULV4	CORO1C	18.46	8.44	10	5	5	8	2.688E6	474
X6RM00	ERC1	18.21	3.48	6	3	3	8	2.271E6	976
E9PJT3	SF3B2	18.14	9.89	5	2	2	5	4.356E6	273
A0A0B4J220	C11orf98	18.12	21.14	3	2	2	5	3.806E6	123
J3QL05	SRSF2	18.11	19.23	4	2	2	7	9.665E6	130
P16615	ATP2A2	17.83	6.81	4	5	5	8	4.316E6	1042
Q86V81	ALYREF	17.71	11.28	2	2	2	6	3.763E6	257
Q5SZU1	PHGDH	17.60	3.01	2	1	1	6	6.093E6	499
P00338	LDHA	17.41	8.13	5	2	3	7	3.832E6	332
F8VPD4	CAD	17.38	1.30	2	2	2	7	2.097E6	2162
H0Y4R1	IMPDH2	17.36	11.91	3	4	4	8	6.849E6	470
P62913	RPL11	17.19	17.42	3	3	3	9	6.223E7	178
A0A087WUT6	EIF5B	17.10	3.85	2	4	4	9	3.824E6	1220
F8W1R7	MYL6	17.09	24.83	12	3	3	8	1.805E6	145
A0A0B4J1Z1	SRSF7	17.05	25.55	3	2	3	7	2.353E7	137
Q99832	CCT7	17.01	7.00	2	3	3	7	4.792E6	543
P27348	YWHAQ	16.98	25.31	8	3	5	8	4.620E6	245
P07195	LDHB	16.94	9.58	8	2	3	6	3.504E6	334
F5GXG4	PGAM5	16.86	15.71	2	2	2	7	3.550E6	140
P20042	EIF2S2	16.31	5.41	1	2	2	9	2.637E6	333
P60842	EIF4A1	16.26	12.32	13	4	4	9	5.339E6	406
Q9Y5A9	YTHDF2	15.98	6.91	3	3	3	7	2.536E6	579
Q9BVP2	GNL3	15.91	13.48	4	6	6	9	4.285E6	549
I3L1P8	SLC25A11	15.87	5.41	2	1	1	4	2.011E6	296
P11586	MTHFD1	15.67	3.96	2	3	3	7	2.894E6	935
E9PD53	SMC4	15.54	4.20	2	5	5	9	1.609E6	1263
Q14152	EIF3A	15.46	5.43	1	6	6	10	3.295E6	1382
P62633	CNBP	15.41	8.47	1	1	1	5	7.423E6	177
P50416	CPT1A	15.29	3.49	3	2	2	5	6.300E6	773
B1AN99	PRSS3	15.03	7.34	2	1	1	8	7.286E6	177
A0A087WUF6	FGF2	14.98	6.25	2	2	2	9	2.884E6	288
Q9H0U4	RAB1B	14.79	30.35	54	5	5	7	9.617E6	201
P25705	ATP5A1	14.68	4.16	6	2	2	5	7.361E6	553
Q13243	SRSF5	14.67	8.09	1	2	2	5	4.148E6	272
P19474	TRIM21	14.56	13.05	2	6	6	10	8.881E6	475
P00367	GLUD1	14.46	8.96	2	4	4	6	3.340E6	558

H7C3D3	ZYX	14.29	30.11	3	3	3	7	1.559E6	176
Q8WX93	PALLD	14.24	0.87	1	1	1	5	2.207E6	1383
P43490	NAMPT	14.12	4.68	2	2	2	8	1.316E6	491
P13674	P4HA1	14.01	4.49	1	2	2	5	1.476E6	534
H0Y4Y4	EFHD2	13.89	12.85	6	2	2	5	4.005E6	179
P23588	EIF4B	13.89	10.97	8	4	4	4	5.481E6	611
J3KTJ1	MYL12A	13.57	34.21	5	3	3	5	5.644E6	114
Q9Y230	RUVBL2	13.44	10.15	4	4	4	6	6.341E6	463
O95782	AP2A1	13.38	4.71	1	4	4	10	2.130E6	977
O60884	DNAJA2	13.26	5.83	1	2	2	6	2.261E6	412
P09234	SNRPC	13.24	7.55	2	1	1	4	2.616E6	159
H0Y9Q7	CANX	13.16	16.47	11	2	2	6	5.738E6	170
Q14681	KCTD2	13.14	14.83	2	2	2	5	1.676E6	263
J3QLI9	SNRPD1	13.04	17.33	2	1	1	4	2.364E6	75
F5GZS6	SLC3A2	13.03	9.02	7	4	4	6	3.799E6	599
P62937	PPIA	12.96	30.30	10	5	5	9	8.334E6	165
Q92504	SLC39A7	12.93	2.99	1	1	1	4	2.375E7	469
A0A087X0P6	IGKV2D-29	12.88	12.75	9	1	1	4	6.562E8	102
P46779	RPL28	12.67	25.55	4	4	4	7	9.170E6	137
P60981	DSTN	12.66	14.55	2	2	2	5	4.848E6	165
P53621	COPA	12.60	3.10	1	3	3	5	3.373E6	1224
P04083	ANXA1	12.59	10.98	3	2	2	4	4.057E6	346
P14625	HSP90B1	12.48	6.10	4	3	4	6	1.149E7	803
P41250	GARS	12.46	2.98	1	2	2	5	1.686E6	739
Q14247	CTTN	12.46	8.55	3	3	3	5	4.271E5	550
P46783	RPS10	12.44	29.09	4	5	5	8	4.536E7	165
P40222	TXLNA	12.39	5.31	1	2	2	5	1.510E6	546
P09543	CNP	12.33	3.33	1	1	1	4	6.266E6	421
D6RAA6	TMEM33	12.22	9.91	3	2	2	4	6.180E6	222
G3V5X6	HNRNPC	12.08	33.33	20	3	3	4	5.564E6	117
Q16527	CSRP2	12.07	12.95	3	2	2	5	2.048E6	193
Q13200	PSMD2	12.02	5.51	3	3	3	5	1.835E6	908
F5H2T0	IKBKAP	12.02	4.58	3	3	3	5	1.140E6	983
Q9NR30	DDX21	12.00	3.45	3	2	2	5	2.282E6	783
F8WBJ6	PNO1	11.99	11.76	2	1	1	4	6.878E5	136
A0A087WX58	HDGFRP2	11.73	22.06	3	1	1	4	3.174E6	68

F5GY37	PHB2	11.58	14.61	7	3	3	4	3.387E6	267
E9PLP0	CARS	11.52	38.28	3	3	4	6	3.693E6	128
Q9Y5M8	SRPRB	11.50	12.92	2	2	2	4	1.530E6	271
P48444	ARCN1	11.46	6.46	3	3	3	5	1.060E6	511
P49207	RPL34	11.43	23.08	1	3	3	5	8.816E6	117
P40227	CCT6A	11.30	12.99	3	5	5	7	5.231E6	531
Q9H0U3	MAGT1	11.26	5.97	2	2	2	5	2.793E6	335
A0A140T933	HLA-A	10.79	12.37	35	2	2	6	3.677E6	299
H0Y7R7	NT5E	10.77	4.78	2	1	1	5	1.852E6	230
H0YFX9	H2AFJ	10.74	20.65	13	1	1	3	2.087E7	92
Q07666	KHDRBS1	10.57	10.84	1	3	3	4	5.471E6	443
P49721	PSMB2	10.45	7.46	1	1	1	4	3.130E6	201
F8VSL7	STOM	10.30	11.31	2	1	1	2	3.312E6	168
P42766	RPL35	10.25	17.89	2	2	2	8	2.295E7	123
Q15029	EFTUD2	10.22	2.37	3	1	2	4	4.216E6	972
D6RBK0	PHB	10.17	17.74	6	2	2	4	2.091E6	124
P62805	HIST1H4A	10.13	29.13	1	3	3	6	1.926E6	103
P00558	PGK1	10.07	3.60	2	1	1	4	5.061E6	417
P60866	RPS20	10.03	19.33	4	2	2	7	3.113E7	119
O14974	PPP1R12A	10.00	4.27	6	3	3	3	7.932E5	1030
P60953	CDC42	9.99	19.90	1	3	3	5	2.947E6	191
P04844	RPN2	9.90	13.15	5	4	4	8	4.498E6	631
H7BXY3	DHX30	9.85	2.06	2	2	2	5	1.467E6	1166
Q14432	PDE3A	9.84	2.28	1	2	2	4	2.101E6	1141
H3BM18	IFI16	9.81	9.58	3	1	1	3	1.283E6	167
A0A0B4J1R6	TKT	9.63	10.72	3	3	3	6	2.241E6	457
P15924	DSP	9.61	0.98	1	3	3	5	4.335E6	2871
J3QQM1	PSMC5	9.54	4.94	2	1	1	3	9.437E5	263
O15084	ANKRD28	9.35	2.94	2	2	2	3	6.473E5	1053
B0QYA4	EIF3D	9.27	13.33	3	1	1	3	4.770E6	105
K7ES31	EIF3K	9.22	8.03	3	1	1	6	5.054E6	137
G5E9W7	MRPS22	9.18	6.90	4	2	2	4	2.227E6	319
I3L3P7	RPS15A	9.14	32.00	8	3	3	6	2.303E6	100
P12259	F5	9.12	0.58	2	1	1	3	6.798E6	2224
J3QT28	BUB3	9.01	5.04	2	1	1	3	3.396E6	278
H7BY36	EWSR1	8.99	7.47	5	2	2	6	4.064E6	308

Q02413	DSG1	8.96	1.53	1	1	1	3	1.313E6	1049
Q5T1S7	PPP6C	8.94	27.20	2	2	2	3	2.118E6	125
Q9Y262	EIF3L	8.94	4.43	6	2	2	3	3.193E6	564
D6R9P3	HNRNPAB	8.90	8.57	4	1	2	3	6.989E6	280
M0QXC3	TIMM50	8.89	15.32	8	1	1	3	1.272E6	111
P31327	CPS1	8.85	3.80	1	4	4	5	1.986E6	1500
Q96GA3	LTV1	8.81	4.63	2	2	2	5	1.564E6	475
Q2NL82	TSR1	8.79	2.49	1	2	2	4	2.577E6	804
Q13347	EIF3I	8.79	12.31	2	3	3	4	6.125E5	325
P20290	BTF3	8.69	22.82	2	1	2	3	9.615E6	206
M0QXS5	HNRNPL	8.49	10.38	4	4	4	6	3.407E6	530
P61981	YWHAG	8.47	11.34	7	1	3	4	3.622E6	247
H7BY82	COL5A1	8.46	13.81	3	2	2	3	1.374E6	210
P62899	RPL31	8.45	26.40	6	3	3	3	4.197E7	125
P28074	PSMB5	8.41	9.13	2	2	2	4	5.624E5	263
Q9NZB2	FAM120A	8.32	3.40	3	3	3	4	1.817E6	1118
K7EJT5	RPL22	8.26	51.06	7	2	2	3	3.803E7	47
A0A087X243	GSTP1	8.23	28.99	2	1	1	3	7.516E6	69
A0A0M3HER1	LIMS1	8.23	5.74	3	1	1	3	3.513E6	209
E9PKH2	SERPINH1	8.10	5.47	2	1	1	3	3.176E6	201
E7ENM1	STAT1	8.03	11.33	5	1	1	2	1.778E6	150
P18077	RPL35A	8.02	14.55	4	2	2	5	2.315E6	110
Q8WXE9	STON2	7.92	0.99	1	1	1	3	4.902E6	905
Q01650	SLC7A5	7.87	6.31	1	2	2	3	1.162E6	507
O95347	SMC2	7.81	3.09	3	3	3	4	2.174E6	1197
Q15645	TRIP13	7.71	8.56	1	3	3	3	4.067E6	432
O43242	PSMD3	7.66	4.12	1	2	2	3	2.945E6	534
K7EM20	YWHAE	7.65	34.78	5	2	3	3	3.696E6	115
P13010	XRCC5	7.59	4.64	1	2	2	4	3.728E6	732
P31946	YWHAB	7.57	13.01	11	1	3	3	3.621E6	246
Q14192	FHL2	7.43	11.11	3	3	3	4	2.019E6	279
Q9BWF3	RBM4	7.41	14.84	3	3	3	4	1.524E6	364
X6RJP6	TAGLN2	7.40	12.30	2	2	2	3	4.237E6	187
Q5HY57	EMD	7.37	10.05	2	2	2	4	1.837E6	219
A0A087WYW6	CCDC47	7.33	5.26	2	1	1	3	1.539E6	190
O00178	GTPBP1	7.32	3.59	3	2	2	5	2.516E7	669

C9J338	TNIK	7.25	3.71	15	1	1	3	1.485E6	350
Q5H907	MAGED2	7.20	4.03	6	2	2	3	2.562E6	521
E9PF19	TBL2	7.09	6.08	4	2	2	2	4.121E6	411
Q15404	RSU1	7.04	13.36	1	3	3	3	1.419E6	277
P28066	PSMA5	7.02	5.39	1	1	1	3	4.807E6	241
P17931	LGALS3	6.93	4.40	1	1	1	3	1.646E6	250
E9PH82	FAM98A	6.87	4.49	2	1	1	2	3.023E6	312
X6R6Z1	ILF2	6.85	12.17	3	1	1	2	8.699E6	115
A0A087WY61	NUMA1	6.82	1.29	6	2	2	2	2.352E5	2099
Q00610	CLTC	6.78	3.64	2	4	4	5	1.923E6	1675
Q8TEQ6	GEMIN5	6.66	1.86	1	2	2	3	1.465E6	1508
O60762	DPM1	6.64	10.38	3	2	2	2	4.560E6	260
Q8WXX5	DNAJC9	6.61	4.62	1	1	1	3	3.120E6	260
H3BRG4	UQCRC2	6.55	8.50	5	2	2	2	1.793E6	412
Q92900	UPF1	6.49	1.86	1	2	2	3	2.666E6	1129
P04004	VTN	6.44	3.14	1	1	1	2	1.400E7	478
Q13155	AIMP2	6.44	11.56	3	2	2	2	5.801E6	320
F8VQ14	CCT2	6.43	2.88	3	1	1	3	3.811E6	416
E7EX73	EIF4G1	6.42	2.37	9	2	2	2	1.857E6	1436
F8WAG2	NEK7	6.42	12.20	2	1	1	3	2.930E6	82
P78527	PRKDC	6.41	0.61	1	2	2	4	2.827E6	4128
H3BV41	NUDT21	6.36	23.94	3	1	1	2	2.091E6	71
Q09666	AHNAK	6.27	3.36	1	2	3	5	2.865E6	5890
Q9H4F8	SMOC1	6.25	2.76	1	1	1	4	1.090E6	434
B4DXZ6	FXR1	6.25	6.91	13	3	3	3	3.066E6	608
Q8WU90	ZC3H15	6.21	2.82	1	1	1	2	1.350E6	426
H7C5S7	RFC2	6.21	46.43	7	1	1	2	2.011E6	28
A0A0G2JI50	NELFE	6.20	8.33	5	1	1	3	3.753E6	240
D6RBF8	SRPK1	6.17	10.96	3	1	1	2	3.097E6	146
Q01780	EXOSC10	6.14	1.47	1	1	1	2	2.281E6	885
P02458	COL2A1	6.09	0.67	1	1	1	3	6.776E6	1487
Q5RKV6	EXOSC6	5.95	5.88	1	1	1	2	1.915E6	272
D6RIY6	EXOSC9	5.94	5.20	2	2	2	4	2.108E6	423
M0R061	COPE	5.93	13.70	3	1	1	2	3.017E6	146
P40938	RFC3	5.90	7.87	2	2	2	2	1.331E6	356
P60174	TPI1	5.85	8.74	2	2	2	2	1.260E6	286

H0YAW4	EIF3E	5.81	19.23	3	2	2	3	1.280E6	156
G3V1J5	DIS3	5.76	1.88	2	1	1	2	5.974E6	796
Q5D862	FLG2	5.75	0.50	1	1	1	4	2.662E6	2391
Q9HCE0	EPG5	5.66	0.35	1	1	1	3	4.938E6	2579
C9JA28	SSR3	5.61	8.05	2	1	1	2	3.780E6	174
A0A0J9YXL8	OCA2	5.60	1.38	3	1	1	4	1.083E7	724
H9KV85	NIN	5.58	1.20	5	1	2	2	3.988E7	1581
Q9BYK8	HELZ2	5.47	0.79	1	2	2	2	9.237E5	2649
P09914	IFIT1	5.43	2.30	1	1	1	2	1.642E6	478
Q5QNZ2	ATP5F1	5.35	6.15	2	1	1	2	1.025E6	195
P36542	ATP5C1	5.34	4.03	1	1	1	2	5.544E6	298
Q15459	SF3A1	5.32	1.51	1	1	1	2	1.120E6	793
P52815	MRPL12	5.31	6.06	2	1	1	2	1.845E6	198
Q9H223	EHD4	5.26	2.77	1	1	1	2	6.591E5	541
A0A1B0GWA2	AGPS	5.24	2.21	2	1	1	2	1.248E6	543
E9PL69	RRM1	5.19	2.63	2	1	1	2	1.460E6	570
Q6P1N7	TAPBP	5.09	8.33	7	1	1	3	2.421E6	156
B8ZZZ7	SPATS2L	5.01	2.41	2	1	1	3	3.157E5	498
P31153	MAT2A	4.97	2.78	1	1	1	2	1.745E6	395
C9J0E4	CSTA	4.96	19.05	2	1	1	2	9.652E5	63
J3QSV6	RSL1D1	4.92	3.02	2	1	1	2	1.044E6	430
K7EQG1	QPCTL	4.92	9.09	2	1	1	2	2.337E6	121
H0Y394	HDLBP	4.87	1.23	3	1	1	2	1.312E6	973
A0A0A0MS41	SFXN3	4.85	4.67	2	1	1	2	2.695E6	321
E9PI21	HSD17B12	4.77	8.54	3	1	1	2	3.835E6	164
G5E9Z2	CLPTM1L	4.74	3.52	2	1	1	2	1.274E6	369
P06730	EIF4E	4.71	10.14	2	2	2	2	6.572E5	217
Q9H0S4	DDX47	4.69	2.42	1	1	1	2	8.308E5	455
Q9Y305	ACOT9	4.67	2.51	1	1	1	2	9.058E5	439
O75475	PSIP1	4.65	1.51	1	1	1	3	2.406E8	530
X6RAC9	EIF1AX	4.65	8.62	4	1	1	2	1.578E6	116
P50991	CCT4	4.64	5.94	1	2	3	3	1.860E6	539
Q9UKD2	MRTO4	4.64	10.46	1	2	2	4	2.302E6	239
A0A087WW69	EIF2B4	4.63	6.14	5	1	1	2	1.148E6	228
Q9Y3I0	RTCB	4.62	4.75	1	2	2	3	2.792E6	505
F8WF48	SEC62	4.61	14.12	3	1	1	2	9.899E5	85

K7ENM2	NCLN	4.54	5.18	3	1	1	2	1.658E6	328
E9PQP7	PPP6R3	4.52	3.74	7	2	2	2	1.479E6	641
B4DZG7	ARL1	4.48	17.04	3	2	2	2	7.792E5	135
E5RHC7	EIF3H	4.44	41.30	8	1	1	1	1.158E6	46
P17812	CTPS1	4.40	1.69	1	1	1	2	8.291E5	591
Q5JRC6	PHF6	4.36	4.94	3	1	1	1	1.023E6	324
B8ZZS4	ANKZF1	4.31	5.04	2	2	2	2	5.511E6	516
Q9Y295	DRG1	4.31	4.09	1	1	1	2	1.153E5	367
P51148	RAB5C	4.23	10.65	11	2	2	2	8.438E5	216
Q53EP0	FNDC3B	4.18	1.08	1	1	1	2	8.093E5	1204
O43147	SGSM2	4.17	0.80	1	1	1	2	5.398E6	1006
E9PQ63	CBR1	4.14	8.99	4	1	1	1	4.329E5	178
J3KT19	USP10	4.12	6.73	2	1	1	2	3.344E6	208
A0A0U1RQV4	ROCK1	4.07	2.02	2	2	2	2	1.056E6	1137
H3BNP9	SQRDL	4.07	10.48	2	1	1	2	5.177E6	124
E9PGF6	PHLDB2	4.04	1.21	3	1	1	3	4.833E6	744
P23246	SFPQ	3.99	3.68	1	2	2	2	8.576E6	707
Q92974	ARHGEF2	3.91	2.13	6	2	2	2	1.694E6	986
A8MUF7	HBE1	3.87	11.49	10	1	1	2	1.489E7	87
H0YLR3	SNRPA1	3.84	13.48	3	1	1	2	1.483E6	89
C9JIG9	OXSR1	3.83	3.21	2	1	1	1	4.017E5	468
P31948	STIP1	3.76	1.84	1	1	1	3	2.257E6	543
O95084	PRSS23	3.75	5.22	1	2	2	2	1.125E6	383
Q9BRP8	PYM1	3.74	8.82	1	1	1	1	9.597E5	204
G3XAN4	TRAM1	3.65	2.43	2	1	1	2	1.180E6	288
V9GYJ9	DAP3	3.63	5.26	3	1	1	1	2.689E6	266
P48047	ATP5O	3.63	5.16	2	1	1	2	1.054E7	213
Q01081	U2AF1	3.58	8.75	1	2	2	3	7.201E6	240
O75643	SNRNP200	3.54	0.94	1	2	2	2	4.836E5	2136
P26639	TARS	3.52	3.04	1	2	2	2	1.022E6	723
Q5JR08	RHOC	3.39	4.26	3	1	1	3	2.639E6	188
H0Y919	RIOK2	3.39	11.43	2	1	1	1	2.437E6	140
O43819	SCO2	3.36	3.76	1	1	1	2	1.540E6	266
K7EN42	NMT1	3.34	14.77	3	1	1	1	7.558E5	88
O00299	CLIC1	3.26	7.47	1	1	1	1	2.295E6	241
A0A087X074	SPIN2A	3.25	16.56	5	2	2	2	4.618E5	157

D6RH20	MRPS27	3.24	4.68	3	1	1	1	1.087E6	299
O95816	BAG2	3.23	5.21	1	1	1	1	3.689E6	211
A0A0D9SFS2	TRIP6	3.20	6.19	2	1	1	1	1.999E6	339
Q9BQE5	APOL2	3.18	3.26	2	1	1	1	8.979E5	337
E7EPW2	MRPS25	3.13	11.82	2	1	1	1	6.027E5	110
Q99683	MAP3K5	3.12	1.02	1	1	1	1	2.480E5	1374
P08240	SRPRA	3.10	2.04	1	1	1	1	1.083E6	638
P31942	HNRNPH3	3.07	3.47	1	1	1	1	2.249E6	346
H7C3P7	RALA	2.96	8.54	2	1	1	1	5.635E6	164
Q15125	EBP	2.90	5.22	1	1	1	1	1.436E6	230
E5RJR5	SKP1	2.85	7.36	2	1	1	1	1.806E6	163
Q9Y2W1	THRAP3	2.84	1.36	1	1	1	1	8.112E5	955
Q8TDB6	DTX3L	2.83	1.76	1	1	1	1	5.724E5	740
D6RIC3	NOP16	2.79	12.00	4	1	1	1	1.633E6	150
X6RL62	OMA1	2.77	3.17	5	1	1	1	8.403E6	252
D6RJ96	HSPA4L	2.74	2.64	5	1	1	1	2.235E6	531
H0Y586	PSMA7	2.74	8.02	2	1	1	1	6.582E5	187
Q8N1F7	NUP93	2.73	3.30	7	2	2	2	1.808E6	819
D6RCQ0	EEF1E1	2.72	11.70	5	1	1	1	2.278E6	94
F8WJN3	CPSF6	2.72	2.93	2	1	1	1	4.797E6	478
P27694	RPA1	2.71	2.60	1	1	1	1	1.636E5	616
Q3KQU3	MAP7D1	2.66	1.90	1	1	1	1	1.075E6	841
E9PRZ0	MAP2K3	2.66	6.88	3	1	1	1	9.497E5	160
Q8IWX8	CHERP	2.65	1.53	2	1	1	1	1.732E6	916
M0QXD6	GTF2F1	2.64	3.70	2	1	1	1	1.874E6	433
Q9UDY2	TJP2	2.61	1.01	2	1	1	1	1.098E6	1190
J3KQ96	TCOF1	2.61	1.06	3	1	1	1	3.046E5	1414
Q9UBV8	PEF1	2.61	6.34	1	1	1	1	2.724E5	284
G3V3U4	PSMA6	2.60	12.15	5	1	1	1	8.950E5	107
K7ELA4	CBX1	2.57	11.59	5	1	1	1	1.496E6	138
C9JD14	GNB4	2.57	12.66	16	1	1	1	6.258E6	79
A3KFL1	EXOSC2	2.57	6.00	4	1	1	1	1.259E6	200
H0YKB1	TJP1	2.56	3.13	5	1	1	1	3.209E5	383
H0YET2	CD59	2.54	23.08	4	1	1	1	9.322E5	52
B4DP62	SLC25A1	2.54	5.29	2	1	1	1	1.566E6	208
P17987	TCP1	2.52	3.96	8	2	2	2	1.647E6	556

P00403	MT-CO2	2.52	7.05	1	1	1	1	3.594E7	227
H0Y7C1	ATP1A2	2.52	1.40	4	1	1	1	2.964E5	714
P02765	AHSG	2.49	3.27	1	1	1	1	3.779E6	367
P61224	RAP1B	2.49	17.93	22	3	3	4	1.713E6	184
Q14978	NOLC1	2.48	2.00	2	1	1	1	2.759E5	699
P62318	SNRPD3	2.48	7.94	1	1	1	1	1.701E7	126
Q15293	RCN1	2.47	3.32	1	1	1	1	3.262E6	331
K7EN53	SRP68	2.47	33.33	3	1	1	1	1.390E5	54
Q8NG97	OR2Z1	2.47	7.64	1	1	1	1	6.497E6	314
Q5SWX8	ODR4	2.47	3.08	1	1	1	1	8.521E6	454
P51665	PSMD7	2.46	3.09	1	1	1	1	1.024E6	324
Q9UMS4	PRPF19	2.46	4.56	1	1	1	1	2.397E6	504
C9K0U8	SSBP1	2.45	12.40	4	1	1	1	6.641E5	121
P0C221	CCDC175	2.43	1.13	2	1	1	1	3.834E7	793
P35625	TIMP3	2.42	5.69	1	1	1	1	2.260E6	211
Q9UBX3	SLC25A10	2.42	3.48	2	1	1	2	7.027E5	287
B8ZZ10	LUC7L	2.41	6.59	5	1	1	1	1.201E6	182
E9PRR7	FRG1	2.39	13.79	2	1	1	1	1.823E6	116
O75746	SLC25A12	2.39	1.77	1	1	1	1	3.611E5	678
G3V361	CALM1	2.38	16.33	6	1	1	1	6.741E6	98
P81605	DCD	2.36	10.00	1	1	1	1	3.412E6	110
Q15393	SF3B3	2.36	1.15	1	1	1	1	5.588E6	1217
H3BQZ7	HNRNPUL2-BS	2.35	1.74	2	1	1	1	2.832E6	746
A0A087WTG3	CUL3	2.35	5.56	1	1	1	1	5.536E5	342
P55884	EIF3B	2.33	1.11	1	1	1	1	2.902E5	814
C9JDV8	NIPSNAP1	2.33	12.00	3	1	1	1	2.305E6	75
I3L0S0	P4HB	2.32	8.11	4	1	1	1	2.476E6	148
I3L3Q7	C1QBP	2.32	7.34	3	1	1	1	2.131E6	177
A0A0U1RQH7	RBM39	2.32	4.08	4	1	1	1	9.432E5	245
Q9H0U6	MRPL18	2.32	7.22	1	1	1	1	5.512E5	180
Q12834	CDC20	2.31	2.40	1	1	1	1	2.907E5	499
I3L3X7	PSMB6	2.29	9.80	3	1	1	1	7.723E5	102
J3QRY4	PSMD11	2.28	6.95	2	1	1	1	6.716E5	187
E9PS68	PC	2.28	4.70	2	1	1	1	2.365E6	298
D6RD66	WDR1	2.26	6.53	2	1	1	1	7.803E5	245
E9PLG2	PSMC3	2.24	4.89	7	1	1	1	7.468E5	184

D6RE77	RAPGEF6	2.24	0.54	4	1	1	1	8.654E5	1114
H0YJI5	RAD51B	2.24	17.14	3	1	1	1	9.323E6	35
E7EW37	TNPO1	2.21	8.09	2	1	1	1	1.388E6	136
Q6DKI1	RPL7L1	2.20	3.66	1	1	1	2	7.166E6	246
H7C2X7	LSG1	2.20	3.86	2	1	1	1	2.487E5	285
P38919	EIF4A3	2.19	2.68	1	1	1	1	7.479E6	411
E5RHP0	NME1	2.18	12.20	5	1	1	1	2.065E6	82
D6RDG3	BTF3	2.18	26.61	2	1	2	2	8.276E5	109
Q7Z417	NUFIP2	2.18	1.87	1	1	1	1	7.735E5	695
E9PH38	PPP2R1A	2.18	22.73	3	2	2	2	2.974E6	132
H7BYY8	PES1	2.15	7.41	4	1	1	1	2.448E5	135
F2Z2W6	HMGN1	2.14	29.55	4	1	1	1	2.314E5	44
S4R414	PITPNM2	2.14	1.67	2	1	1	1	2.765E6	478
O15460	P4HA2	2.11	2.24	1	1	1	1	1.968E6	535
P51857	AKR1D1	2.11	3.07	1	1	1	2	8.390E6	326
E9PS97	PARVA	2.10	6.70	3	1	1	1	6.664E5	209
P32455	GBP1	2.08	1.35	1	1	1	1	1.965E6	592
E9PLA7	NXF1	2.04	13.04	4	1	1	1	1.734E6	115
F8W7L6	GRIA2	2.03	2.74	7	1	1	1	2.233E6	730
H0Y8N7	MRPL1	2.01	5.19	2	1	1	1	6.530E5	231
A0A087WTS8	HSPA4	1.99	2.95	3	1	1	1	1.513E6	475
P00846	MT-ATP6	1.98	4.42	1	1	1	2	2.432E6	226
Q96GM8	TOE1	1.98	2.94	1	1	1	1	7.179E5	510
E7ET15	U2SURP	1.97	2.72	4	2	2	2	3.405E6	1028
Q5JR04	MOV10	1.96	1.37	2	1	1	1	1.384E6	947
Q5T749	KPRP	1.96	1.90	1	1	1	1	2.131E5	579
Q06210	GFPT1	1.96	1.86	1	1	1	1	1.309E6	699
H7C5R5	EIF2A	1.95	5.38	3	1	1	1	1.560E6	316
P42285	SKIV2L2	1.94	1.06	1	1	1	1	1.127E7	1042
Q7L5L3	GDPD3	1.93	2.20	1	1	1	1	4.770E7	318
Q13228	SELENBP1	1.89	1.69	1	1	1	1	4.964E6	472
M0R0P1	FBL	1.88	13.16	9	2	2	2	1.907E6	228
O43852	CALU	1.87	6.67	2	2	2	3	2.571E6	315
P61619	SEC61A1	1.83	2.31	2	1	1	1	1.535E6	476
Q9NTK5	OLA1	1.79	2.02	2	1	1	1	3.453E7	396
E7ERY9	ATP2B1	1.79	1.14	6	1	1	1	3.014E5	963

P0C6C1	ANKRD34C	1.77	2.80	1	1	1	1	3.587E6	535
B4DPS1	STK16	1.74	3.21	4	1	1	1	1.070E7	187
Q8TAK6	OLIG1	1.74	4.43	1	1	1	1	3.501E7	271
E9PC52	RBBP7	1.72	2.40	2	1	1	1	1.374E6	416
A0A0D9SF19	TRIM56	1.71	10.77	3	1	1	1	5.479E5	65
Q9H0U9	TSPYL1	1.71	3.89	1	1	1	1	1.828E6	437
C9JZR2	CTNND1	1.68	1.28	2	1	1	1	6.898E5	938
O14802	POLR3A	1.67	0.50	1	1	1	1	1.735E6	1390
Q9BSD7	NTPCR	1.67	14.21	2	2	2	2	1.207E6	190
D6R997	COPB2	1.65	7.89	2	1	1	2	1.171E6	152
Q49AG3	ZBED5	1.64	2.16	1	1	1	1	4.917E7	693
Q8TCJ2	STT3B	1.63	1.45	1	1	1	3	5.301E6	826
F5H0N7	CLIP1	1.61	0.89	3	1	1	1	4.954E6	1013
K7EQ46	ZNF540	1.61	7.00	3	1	1	1	2.291E6	100
B1AM21	GNAQ	0.00	8.82	4	1	1	1	1.655E5	170
C9J8D4	MOGS	0.00	3.67	2	1	1	2	4.952E6	491
C9JG86	SETD2	0.00	3.06	3	1	1	1	6.468E6	1340
C9JLV4	APAF1	0.00	1.72	2	1	1	1	1.588E7	1163
C9K0J5	RAPH1	0.00	0.54	1	1	1	1	5.897E6	1302
D6R9X1	CDK18	0.00	9.72	4	1	1	1	2.530E5	72
F5GXS9	WBP11	0.00	16.98	3	1	1	1	1.419E6	53
F5H7A8	CCDC189	0.00	16.90	3	1	1	1	5.985E6	71
F8W7T0	TES	0.00	13.92	2	1	1	1	6.428E5	79
F8WCJ1	EIF5A2	0.00	17.14	8	1	1	1	1.887E6	105
G8JLD5	DNM1L	0.00	2.53	2	1	1	1	1.810E7	712
H0Y5E8	SAR1A	0.00	10.43	2	1	1	3	4.952E6	115
H3BLY5	ISG20	0.00	13.79	3	1	1	2	4.484E5	87
H7BYE4	MSL3	0.00	10.34	1	1	1	1	1.237E6	116
H7C561	SF1	0.00	4.47	2	1	1	1	4.856E5	291
J3KMZ7	INTS2	0.00	1.67	2	1	1	1	9.534E6	1196
J3QQQ6	TOX3	0.00	23.33	1	1	1	1	8.655E6	60
K7EJ19	NARS	0.00	8.82	5	1	1	1	1.335E6	136
O15427	SLC16A3	0.00	2.80	1	1	1	5	4.209E6	465
P05109	S100A8	0.00	11.83	1	1	1	1	4.253E5	93
P11166	SLC2A1	0.00	2.03	1	1	1	1	2.606E6	492
P15328	FOLR1	0.00	2.72	1	1	1	2	2.613E6	257

P25024	CXCR1	0.00	2.29	2	1	1	1	1.572E6	350
P35125	USP6	0.00	0.85	1	1	1	1	2.733E6	1406
P46063	RECQL	0.00	2.62	1	1	1	1	7.944E5	649
P53701	HCCS	0.00	4.10	1	1	1	1	2.495E6	268
P54707	ATP12A	0.00	0.96	1	1	1	1	1.404E5	1039
Q13242	SRSF9	0.00	4.52	1	1	1	1	1.191E6	221
Q3KQZ1	SLC25A35	0.00	5.00	1	1	1	1	1.472E7	300
Q53T94	TAF1B	0.00	5.95	1	1	1	1	4.784E5	588
Q6ZMU1	C3P1	0.00	9.09	1	1	1	2	4.358E5	363
Q7KZF4	SND1	0.00	1.43	1	1	1	1	3.393E6	910
Q7Z5H4	VN1R5	0.00	1.96	1	1	1	1	1.258E7	357
Q8NEK8	FAM46D	0.00	6.43	1	1	1	2	8.863E5	389
Q8WWY6	MBD3L1	0.00	5.15	1	1	1	1	4.987E5	194
Q92538	GBF1	0.00	0.59	1	1	1	1	8.656E5	1859
Q96P31	FCRL3	0.00	2.04	1	1	1	1	1.670E8	734
Q9BZD2	SLC29A3	0.00	4.00	1	1	1	1	1.414E5	475
Q9C0D2	CEP295	0.00	1.04	1	1	1	1	8.594E5	2601
Q9H9Y2	RPF1	0.00	2.58	1	1	1	1	2.895E6	349
Q9NV31	IMP3	0.00	3.80	1	1	1	1	4.368E5	184
Q9UHD8	SEPT9	0.00	1.19	1	1	1	1	1.298E7	586
Q9Y3B7	MRPL11	0.00	5.73	1	1	1	1	7.858E5	192
Q9Y597	KCTD3	0.00	3.44	1	1	1	1	2.492E6	815
Q9Y613	FHOD1	0.00	3.87	1	1	1	1	1.364E7	1164
R4GN19	RPL36A	0.00	32.00	6	1	1	1	7.748E6	25
Q68E03	DKFZ	0.00	4.59	4	1	1	1	1.275E6	283
A0A0A0MSL1	DLG5	0.00	1.25	2	1	1	1	3.012E6	880
B5MED8	SORCS2	0.00	1.32	2	1	1	1	3.766E6	987

Supplementary Table 5: Identification of 164 proteins using LC-MS/MS in PC9 cell.

Accession	Gene Symbol	Score	Coverage	# Proteins	# Unique Peptides	# Peptides	# PSMs	Area	# AAs
Q05639	EEF1A2	768.62	68.25	1	10	20	237	3.096E9	463
P26641	EEF1G	693.97	45.08	1	18	18	250	1.794E9	437
P26640	VARS	614.61	32.52	7	38	38	217	3.697E8	1264
P29692	EEF1D	445.41	59.79	20	6	16	145	2.089E9	281
P68104	EEF1A1	435.48	36.36	6	2	12	128	3.096E9	462
O14744	PRMT5	350.12	35.01	12	17	17	115	3.385E8	637
P60709	ACTB	221.73	43.20	20	4	14	84	5.896E8	375
E9PL71	EEF1D	211.41	48.13	5	1	9	65	1.376E9	187
Q9BQA1	WDR77	185.44	31.29	2	7	7	53	2.252E8	342
P24534	EEF1B2	184.15	52.00	4	5	9	46	3.947E8	225
P52732	KIF11	179.84	14.96	1	16	16	70	2.715E7	1056
P68032	ACTC1	168.44	30.24	13	1	11	60	2.625E8	377
B8ZZF0	PPM1B	153.75	31.35	5	7	7	62	1.078E7	303
H0YA55	ALB	115.53	9.47	8	5	5	39	5.185E7	454
P11142	HSPA8	100.25	22.29	15	9	12	40	3.548E7	646
Q96C19	EFHD2	93.38	25.00	6	6	6	29	2.831E7	240
P68371	TUBB4B	92.72	17.53	25	2	6	26	1.908E7	445
H0Y2Y8	ZYX	70.66	26.67	6	9	9	23	7.100E7	540
P07437	TUBB	69.87	14.86	25	1	5	18	1.419E7	444
P35579	MYH9	64.08	4.54	6	9	9	22	5.897E6	1960
P38646	HSPA9	62.67	5.89	4	3	3	20	7.245E6	679
Q562R1	ACTBL2	62.21	16.22	1	1	5	23	4.934E8	376
P42704	LRPPRC	60.69	8.25	4	10	10	24	9.393E6	1394
A0A0A0MRV0	RRBP1	58.97	8.44	12	11	11	22	8.741E6	1410
P04406	GAPDH	54.77	21.79	2	6	6	24	4.155E7	335
P07900	HSP90AA1	54.00	9.84	7	4	6	19	1.623E7	732
Q06830	PRDX1	46.37	16.58	3	2	2	12	5.565E6	199
P0DMV9	HSPA1B	44.67	11.70	5	4	6	19	3.972E7	641
Q5D862	FLG2	44.23	0.50	1	1	1	16	2.252E6	2391
P07195	LDHB	43.77	15.57	11	3	4	14	1.684E7	334
E9PPU1	RPS3	41.21	34.81	11	4	4	13	8.867E6	158
P05198	EIF2S1	38.34	14.29	3	4	4	15	3.418E6	315

P67809	YBX1	38.21	9.88	5	3	3	13	6.665E6	324
Q15208	STK38	36.65	20.00	1	8	8	16	3.157E7	465
P35637	FUS	35.43	3.04	4	2	2	11	1.082E7	526
O43707	ACTN4	33.90	9.88	18	9	9	18	5.907E6	911
Q5HYB6	DKFZ	33.09	16.81	5	3	3	10	6.135E6	232
J3QLE5	SNRPN	31.99	8.88	3	1	1	8	6.878E6	169
P60842	EIF4A1	31.92	9.61	15	3	3	8	3.757E6	406
P15121	AKR1B1	31.90	24.05	4	6	6	16	2.480E7	316
P08238	HSP90AB1	31.80	8.15	7	3	5	11	1.374E7	724
P68366	TUBA4A	31.07	9.82	23	3	3	14	3.082E7	448
P60174	TPI1	30.58	17.83	4	4	4	10	3.935E6	286
P06733	ENO1	28.32	17.28	7	7	7	14	1.711E7	434
Q15293	RCN1	27.62	7.85	1	2	2	9	3.266E6	331
B4DLR8	NQO1	27.08	20.30	4	3	3	9	5.949E6	202
P11021	HSPA5	26.95	10.40	1	3	5	11	1.983E7	654
E9PG15	YWHAQ	25.68	14.77	5	1	2	6	3.040E6	149
D6RF44	HNRNPD	25.50	12.61	5	1	1	8	6.200E6	111
H3BUH7	ALDOA	25.26	17.42	7	2	2	8	1.195E7	155
A0A087X0P6	IGKV2D-29	24.54	12.75	14	1	1	7	2.465E9	102
P00338	LDHA	24.18	15.96	8	4	5	10	2.855E7	332
Q86YZ3	HRNR	23.68	5.16	1	3	3	10	3.765E6	2850
E9PCY7	HNRNPH1	22.98	6.06	21	2	2	8	6.805E6	429
Q9Y6Y0	IVNS1ABP	21.67	5.92	3	4	4	9	4.618E6	642
P81605	DCD	21.22	10.00	1	1	1	9	1.015E7	110
Q00839	HNRNPU	21.08	1.21	1	1	1	8	1.383E6	825
A0A0U1RQT1	ACAP2	20.52	6.95	5	3	3	11	3.125E6	547
F8VX11	EIF4B	18.58	4.44	3	1	1	8	2.306E6	360
A6NMY6	ANXA2P2	17.94	9.44	19	3	3	6	1.727E7	339
F8WD59	RPSA	16.45	11.21	4	1	1	6	4.384E6	116
F8W0G4	PCBP2	16.10	15.82	12	2	2	6	3.315E6	158
P12004	PCNA	14.97	4.98	1	1	1	6	1.769E6	261
P62258	YWHAE	14.81	9.02	2	2	2	6	6.516E6	255
E5RIU6	CDK1	14.75	7.41	4	1	1	5	2.583E6	189
P05141	SLC25A5	14.50	6.04	4	2	2	7	9.112E6	298
K7EQH4	ATP5A1	13.97	24.32	7	2	2	5	3.643E6	111

D6R9W4	DBN1	12.73	5.05	2	1	1	5	2.066E6	317
P67936	TPM4	12.52	10.89	1	2	2	4	1.729E6	248
P63104	YWHAZ	12.26	10.61	10	2	2	4	1.019E7	245
C9JI87	VDAC1	12.06	6.01	2	1	1	5	2.010E6	183
F8W1N5	NACA	12.05	21.13	7	1	1	3	4.938E6	71
P07814	EPRS	10.87	1.59	2	2	2	4	1.639E6	1512
F8VVM2	SLC25A3	10.40	3.70	2	1	1	4	7.598E6	324
P61981	YWHAG	10.02	7.29	4	1	2	5	1.504E6	247
M0R0P7	RPL18A	9.98	9.49	5	1	1	3	9.918E5	137
Q5T6W2	HNRNPK	9.90	2.64	2	1	1	5	2.160E6	379
P47914	RPL29	9.89	9.43	1	1	1	3	2.514E6	159
E7EPB3	RPL14	9.85	8.87	2	1	1	4	4.658E6	124
E9PD92	G6PD	9.53	6.25	4	1	1	2	3.019E6	256
P07954	FH	9.37	2.75	1	1	1	3	2.206E6	510
E9PMI6	CLNS1A	9.24	14.97	5	2	2	6	1.773E7	167
Q5VU13	VSIG8	9.16	2.90	1	1	1	4	4.230E6	414
B5MCW2	RPL3	8.89	4.41	3	1	1	3	5.165E6	272
P31948	STIP1	8.84	1.84	1	1	1	4	1.201E6	543
H0YDP8	TRIM21	8.81	15.56	2	1	1	3	4.914E6	90
P48681	NES	8.69	0.56	1	1	1	4	1.581E8	1621
H3BRG4	UQCRC2	8.67	3.88	2	1	1	2	2.256E6	412
B1AN99	PRSS3	8.55	7.34	2	1	1	3	2.449E6	177
C9JEH7	RPS4Y1	8.47	8.02	4	3	3	6	2.723E6	262
A0A087X2E9	GSTP1	8.25	26.58	3	2	2	3	1.747E6	79
Q14764	MVP	7.92	2.24	6	2	2	4	1.371E6	893
D6RAS7	RPS3A	7.81	13.60	11	2	2	4	8.854E6	125
E9PKH2	SERPINH1	7.76	5.47	2	1	1	3	3.537E6	201
Q8NC51	SERBP1	7.65	6.62	1	2	2	2	1.252E6	408
P62750	RPL23A	7.55	20.51	6	3	3	3	1.513E6	156
P41091	EIF2S3	7.55	4.03	3	2	2	4	2.430E6	472
P16401	HIST1H1B	7.42	4.87	1	1	1	3	1.738E6	226
G3V1B3	RPL21	7.09	17.24	3	1	1	2	7.222E5	87
Q9Y657	SPIN1	6.90	4.96	1	1	1	3	9.073E5	262
C9J9W2	LASP1	6.86	14.46	3	2	2	5	2.943E6	166
O95831	AIFM1	6.50	1.79	1	1	1	2	1.807E6	613

P28072	PSMB6	6.50	4.60	1	1	1	2	1.369E6	239
F8VZS0	RPLP0	6.28	11.79	12	3	3	4	1.341E7	246
P06748	NPM1	6.20	7.48	2	2	2	2	7.973E6	294
Q9Y5B9	SUPT16H	6.08	1.05	1	1	1	2	1.253E6	1047
P80723	BASP1	5.93	6.17	1	1	1	2	1.048E6	227
F8VR77	PA2G4	5.87	3.87	2	1	1	2	1.025E7	284
A0A0J9YXP8	GPI	5.83	4.10	6	1	1	3	5.119E7	268
S4R3D5	AKR1C3	5.22	5.81	8	1	1	3	6.498E6	155
Q02878	RPL6	4.85	3.82	1	1	1	2	1.610E6	288
P30050	RPL12	4.84	9.70	1	1	1	1	3.835E6	165
D6RHF4	UGDH	4.41	18.87	7	1	1	3	7.767E5	53
P30101	PDIA3	4.38	4.75	2	2	2	2	1.869E6	505
O00299	CLIC1	4.35	4.98	1	1	1	2	2.698E6	241
H0YEN5	RPS2	4.31	16.41	6	3	3	3	2.906E6	195
Q4VY20	YWHAB	3.95	29.73	8	1	2	2	2.801E6	74
H0YH33	STRAP	3.76	13.68	2	1	1	1	1.016E6	95
M0QXF2	ATP1A3	3.60	5.98	8	1	1	1	1.053E6	251
H0YJF9	DLST	3.58	18.92	5	1	1	1	1.863E6	74
H0YLU2	PSME1	3.46	12.28	2	1	1	1	1.059E6	114
C9JLS9	PSMC2	3.39	10.00	2	1	1	1	7.044E5	130
E7EU94	PRPF31	3.38	5.77	5	1	1	1	5.658E5	260
P49411	TUFM	3.36	3.32	1	1	1	1	3.359E6	452
R4GNH1	GOLGA1	2.92	8.33	2	1	1	1	1.870E7	168
A0A087X2D0	SRSF3	2.87	12.63	2	1	1	2	1.793E6	95
M0QZC5	RPS11	2.77	9.32	2	1	1	1	1.973E6	118
H0YB86	PABPC1	2.77	6.67	6	1	1	1	7.375E5	165
X6RJP6	TAGLN2	2.72	5.88	2	1	1	1	1.494E6	187
P52907	CAPZA1	2.71	5.24	1	1	1	1	8.054E5	286
P46783	RPS10	2.69	8.48	2	1	1	1	1.034E6	165
Q5SZU1	PHGDH	2.63	2.20	2	1	1	1	1.757E6	499
H0YC68	GSR	2.61	9.92	2	1	1	1	5.358E5	131
A0A087WWY3	FLNA	2.56	0.69	4	1	1	1	1.618E6	2315
Q9NZI8	IGF2BP1	2.56	2.77	1	1	1	1	1.781E6	577
P46777	RPL5	2.53	4.04	1	1	1	1	2.584E6	297
F5GY37	PHB2	2.52	4.49	3	1	1	1	1.312E6	267

A0A087WYS6	PSMA8	2.42	4.91	3	1	1	1	2.308E6	224
Q13347	EIF3I	2.31	3.69	1	1	1	1	1.315E6	325
J3KSJ0	RPL17	2.30	17.86	10	1	1	1	1.110E7	56
F5H867	SLC3A2	2.24	9.63	6	1	1	1	2.365E6	135
Q96J66	ABCC11	2.23	0.94	1	1	1	1	5.146E6	1382
E9PLA9	CAPRIN1	2.23	6.45	3	1	1	1	5.570E6	186
A0A087WXZ1	TP53	2.22	7.14	8	1	1	2	2.638E6	182
B1AHC9	XRCC6	2.16	1.79	2	1	1	1	5.542E5	559
D6RGK8	RACK1	2.13	14.08	13	1	1	1	5.420E6	71
P10809	HSPD1	2.10	2.09	1	1	1	1	5.989E6	573
Q5T8U3	RPL7A	2.09	5.76	2	1	1	1	2.258E6	191
E9PD99	LRCH3	2.09	1.28	2	1	1	1	6.639E6	623
E7ENZ3	CCT5	1.95	2.06	4	1	1	1	9.417E5	486
H3BU31	RPL4	1.94	6.43	3	1	1	1	3.381E5	171
C9JBA4	LYG2	1.89	6.51	3	1	1	2	7.406E5	169
M0R1V7	UBA52	1.83	14.29	20	1	1	1	1.743E6	63
P16403	HIST1H1C	1.82	5.63	3	1	1	1	7.454E6	213
F6UXX1	SYNCRIP	1.80	6.49	4	1	1	1	1.302E6	185
A0A075B6Z2	TRAJ56	0.00	38.10	1	1	1	1	6.832E6	21
F8W9L6	LRMP	0.00	6.32	4	1	1	1	1.300E8	348
H7C048	SMARCE1	0.00	7.01	5	1	1	1	1.265E7	157
O75460	ERN1	0.00	0.72	1	1	1	1	6.859E5	977
P01859	IGHG2	0.00	2.76	1	1	1	1	3.019E6	326
P27540	ARNT	0.00	1.39	1	1	1	1	5.632E6	789
Q5T3N0	ANXA1	0.00	14.78	3	1	1	1	1.788E6	115
Q9NU22	MDN1	0.00	0.13	1	1	1	5	9.797E6	5596
Q96DT5	DNAH11	0.00	0.16	3	1	1	1	5.934E6	4516

ARRIVE

The ARRIVE Guidelines Checklist

Animal Research: Reporting In Vivo Experiments

Carol Kilkenny[1], William J Browne[2], Innes C Cuthill[3], Michael Emerson[4] and Douglas G Altman[5]

[1]The National Centre for the Replacement, Refinement and Reduction of Animals in Research, London, UK, [2]School of Veterinary Science, University of Bristol, Bristol, UK, [3]School of Biological Sciences, University of Bristol, Bristol, UK, [4]National Heart and Lung Institute, Imperial College London, UK, [5]Centre for Statistics in Medicine, University of Oxford, Oxford, UK.

	ITEM	RECOMMENDATION	Section/ Paragraph
Title	1	Provide as accurate and concise a description of the content of the article as possible.	Title
Abstract	2	Provide an accurate summary of the background, research objectives, including details of the species or strain of animal used, key methods, principal findings and conclusions of the study.	Abstract
**INTRODUCTION**			
Background	3	a. Include sufficient scientific background (including relevant references to previous work) to understand the motivation and context for the study, and explain the experimental approach and rationale.<br>b. Explain how and why the animal species and model being used can address the scientific objectives and, where appropriate, the study's relevance to human biology.	Backgroud paragraphs 1-3<br>Method [illegible]
Objectives	4	Clearly describe the primary and any secondary objectives of the study, or specific hypotheses being tested.	Method [illegible]
**METHODS**			
Ethical statement	5	Indicate the nature of the ethical review permissions, relevant licences (e.g. Animal [Scientific Procedures] Act 1986), and national or institutional guidelines for the care and use of animals, that cover the research.	Method paragraph 5
Study design	6	For each experiment, give brief details of the study design including:<br>a. The number of experimental and control groups.<br>b. Any steps taken to minimise the effects of subjective bias when allocating animals to treatment (e.g. randomisation procedure) and when assessing results (e.g. if done, describe who was blinded and when).<br>c. The experimental unit (e.g. a single animal, group or cage of animals).<br>A time-line diagram or flow chart can be useful to illustrate how complex study designs were carried out.	Method paragraphs 5-6
Experimental procedures	7	For each experiment and each experimental group, including controls, provide precise details of all procedures carried out. For example:<br>a. How (e.g. drug formulation and dose, site and route of administration, anaesthesia and analgesia used [including monitoring], surgical procedure, method of euthanasia). Provide details of any specialist equipment used, including supplier(s).<br>b. When (e.g. time of day).<br>c. Where (e.g. home cage, laboratory, water maze).<br>d. Why (e.g. rationale for choice of specific anaesthetic, route of administration, drug dose used).	Method paragraphs 5-6
Experimental animals	8	a. Provide details of the animals used, including species, strain, sex, developmental stage (e.g. mean or median age plus age range) and weight (e.g. mean or median weight plus weight range).<br>b. Provide further relevant information such as the source of animals, international strain nomenclature, genetic modification status (e.g. knock-out or transgenic), genotype, health/immune status, drug or test naïve, previous procedures, etc.	Method paragraphs 5-6

Housing and husbandry	9	Provide details of:<br>a. Housing (type of facility e.g. specific pathogen free [SPF]; type of cage or housing; bedding material; number of cage companions; tank shape and material etc. for fish).<br>b. Husbandry conditions (e.g. breeding programme, light/dark cycle, temperature, quality of water etc for fish, type of food, access to food and water, environmental enrichment).<br>c. Welfare-related assessments and interventions that were carried out prior to, during, or after the experiment.	Method paragraphs 5-6
Sample size	10	a. Specify the total number of animals used in each experiment, and the number of animals in each experimental group.<br>b. Explain how the number of animals was arrived at. Provide details of any sample size calculation used.<br>c. Indicate the number of independent replications of each experiment, if relevant.	Method paragraphs 5-6
Allocating animals to experimental groups	11	a. Give full details of how animals were allocated to experimental groups, including randomisation or matching if done.<br>b. Describe the order in which the animals in the different experimental groups were treated and assessed.	Method paragraphs 5-6
Experimental outcomes	12	Clearly define the primary and secondary experimental outcomes assessed (e.g. cell death, molecular markers, behavioural changes).	Result paragraph 3
Statistical methods	13	a. Provide details of the statistical methods used for each analysis.<br>b. Specify the unit of analysis for each dataset (e.g. single animal, group of animals, single neuron).<br>c. Describe any methods used to assess whether the data met the assumptions of the statistical approach.	Method paragraph 9
RESULTS			
Baseline data	14	For each experimental group, report relevant characteristics and health status of animals (e.g. weight, microbiological status, and drug or test naïve) prior to treatment or testing. (This information can often be tabulated).	Method paragraphs 5-6
Numbers analysed	15	a. Report the number of animals in each group included in each analysis. Report absolute numbers (e.g. 10/20, not 50%[2]).<br>b. If any animals or data were not included in the analysis, explain why.	Figure Legends paragraph 3
Outcomes and estimation	16	Report the results for each analysis carried out, with a measure of precision (e.g. standard error or confidence interval).	Method paragraph 9
Adverse events	17	a. Give details of all important adverse events in each experimental group.<br>b. Describe any modifications to the experimental protocols made to reduce adverse events.	Result paragraph 3
DISCUSSION			
Interpretation/ scientific implications	18	a. Interpret the results, taking into account the study objectives and hypotheses, current theory and other relevant studies in the literature.<br>b. Comment on the study limitations including any potential sources of bias, any limitations of the animal model, and the imprecision associated with the results[2].<br>c. Describe any implications of your experimental methods or findings for the replacement, refinement or reduction (the 3Rs) of the use of animals in research.	Result paragraph 3
Generalisability/ translation	19	Comment on whether, and how, the findings of this study are likely to translate to other species or systems, including any relevance to human biology.	Discussion paragraph4
Funding	20	List all funding sources (including grant number) and the role of the funder(s) in the study.	Funding&Aut hors'contributi

References:

1. Kilkenny C, Browne WJ, Cuthill IC, Emerson M, Altman DG (2010) Improving Bioscience Research Reporting: The ARRIVE Guidelines for Reporting Animal Research. *PLoS Biol* 8(6): e1000412. doi:10.1371/journal.pbio.1000412
2. Schulz KF, Altman DG, Moher D, the CONSORT Group (2010) CONSORT 2010 Statement: updated guidelines for reporting parallel group randomised trials. *BMJ* 340:c332.
